# Quantifying suicide contagion at population scale

**DOI:** 10.1126/sciadv.adq4074

**Published:** 2024-07-31

**Authors:** Jeffrey Shaman, Sasikiran Kandula, Sen Pei, Marta Galanti, Mark Olfson, Madelyn Gould, Katherine Keyes

**Affiliations:** ^1^ Department of Environmental Health Sciences, Mailman School of Public Health, Columbia University, New York, NY 10032, USA.; ^2^ Columbia Climate School, Columbia University, New York, NY 10025, USA.; ^3^ Department of Epidemiology, Mailman School of Public Health, Columbia University, New York, NY 10032, USA.; ^4^ Department of Psychiatry, Columbia University, New York, NY 10032, USA.

## Abstract

The spread of suicidal behavior among individuals is often described as a contagion; however, rigorous modeling of suicide as a dynamic, contagious process is minimal. Here, we develop and validate a model-inference system depicting suicide ideation and death and use it to quantify the contagion processes in the US associated with two prominent celebrity suicide events: Robin Williams during 2014 and Kate Spade and Anthony Bourdain, which occurred 3 days apart during 2018. We show that both events produced large transient increases of suicide contagion contact rates, i.e., the spread of suicidal thought and behavior, and a period of elevated suicidal ideation in the general population. Our modeling approach provides a framework for quantifying suicidal contagion and better understanding, preventing, and containing its spread.

## Teaser

A model depicting suicide contagion is presented and used to quantify contagion rates following high-profile suicides.

## INTRODUCTION

Suicidal behavior is understood to derive from individual, environmental, and social determinants (*1*). Psychiatric disorders, stressful life events, and access to lethal means are dominant drivers of suicide (*2*–*4*); however, a portion of suicidal ideation has long been attributed to social, or contagious, processes (*5*). That is, proximity to or familiarity with persons who have ideated, attempted, or died of suicide can induce suicidal ideation or attempts among susceptible individuals.

Contagious processes are more typically used to describe infectious disease transmission (*6*) or the spread of information (*7*). For these systems, the spread of a pathogen or idea can be mathematically represented as a nonlinear function of the number of individuals capable of transmitting the contagion and the number of individuals susceptible to “infection.” For suicide, contagion may manifest at local scales, such as clusters of suicide deaths in a school population (*8*). These events have been studied as contagion to better understand the processes allowing suicidal ideation and behavior to spread despite natural barriers against self-harm; knowledge of a suicide attempt has been shown to lead to emotional distress and greater likelihood of suicidal symptoms, regardless of external controls (*9*).

Across larger geographic and population scales, external drivers (economic downturn, political conflict, and pandemic) can lead to suicidal ideation and behavior (*1*). However, dramatizations of suicide, which may normalize or romanticize suicidal behaviors, e.g., *13 Reasons Why*, can also induce ideation and may manifest as a contagion process effected over a large population (*10*). Celebrity suicide events, in particular, due to familiarity or identification with a famous decedent, may also induce or exacerbate suicidal behaviors. The harmful effects of celebrity suicide on ideation and behavior in the broader population, or particular subpopulations, have been reported consistently (*11*, *12*). Response is thought to be modulated by media and social media coverage that may either amplify or diminish suicidal response behaviors, i.e., Werther v. Papageno effects (*13*), but also, in the case of social media, may serve as a record of amplified or transmitted ideation (*14*).

There have been few attempts to dynamically model and quantify suicide contagion. One notable exception modeled competing rates of transition to death or immunity from suicide following an initial suicide death in a local community (*15*). The authors proposed a system in which a portion of the population is susceptible to dying of suicide following the index suicide; however, some, if not many, of these susceptible individuals, presumably due to community support or intervention, will transition to a nonsusceptible, i.e., immune, state before acting on any suicidal impulse. By fitting their model to records documenting three separate, local suicide clusters, the authors estimated that 15 to 30% of the examined populations fell into the initially susceptible category. Some more recent studies of social contagion, including the spread of rumors or suicidal ideation, have leveraged a variety of modeling approaches but have either remained more theoretical (*16*) or not reconciled their models with actual data on suicidality other than online search terms (*17*).

**Fig. 1.**
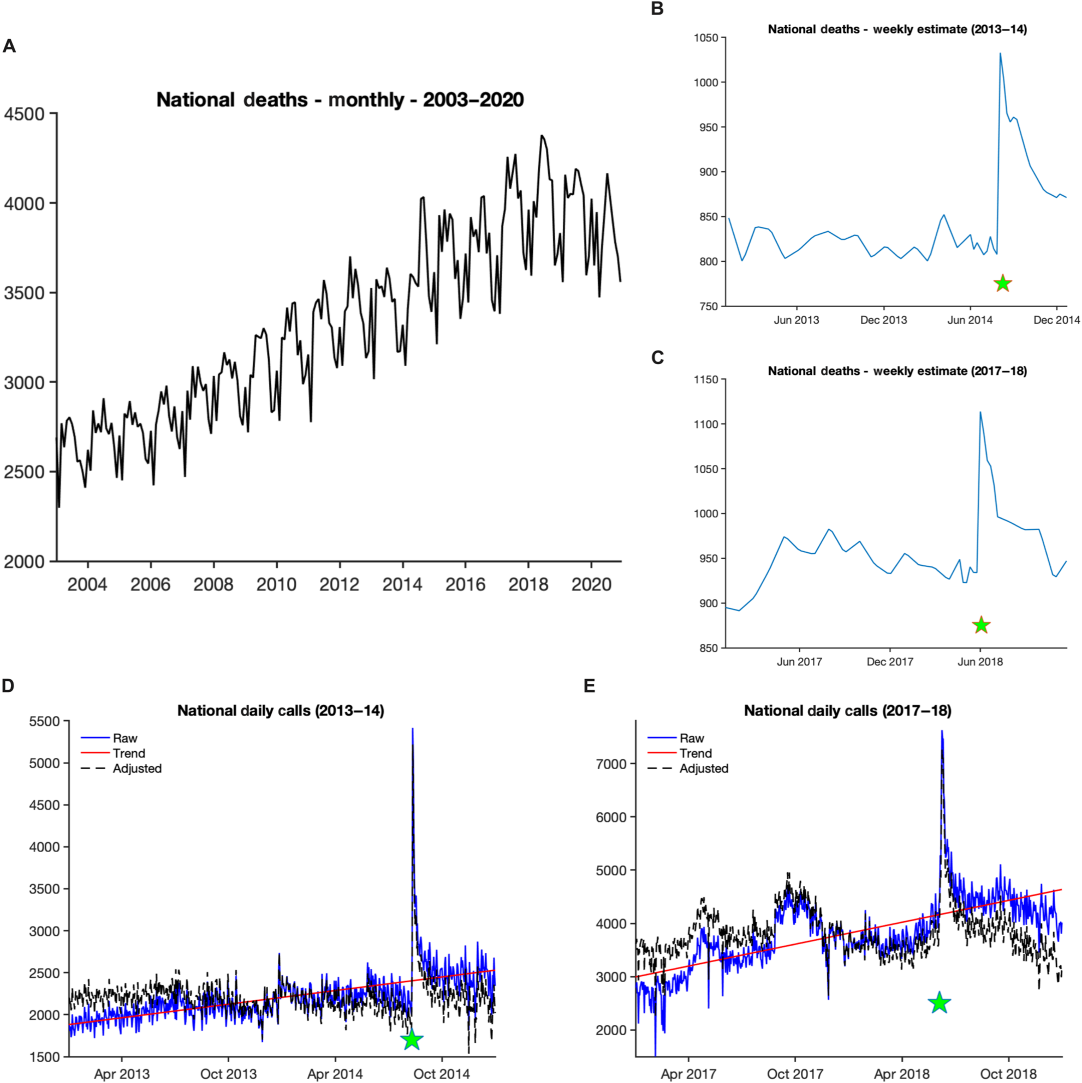
US NVSS suicide deaths and 988 Lifeline calls. (**A**) Monthly national suicide deaths from January 2003 through December 2020. Weekly interpolated estimates of suicide deaths with the seasonal cycle removed for (**B**) 2013–2014 and (**C**) 2017–2018. Daily national calls for (**D**) 2013–2014 and (**E**) 2017–2018; the raw values are the solid blue lines, trends are the red lines, and the detrended values are dashed black lines. Green stars indicate the timing of the celebrity suicide events.

Here, we aim to expand understanding of suicide spread by quantifying suicide contagion rates at US national scales in response to two celebrity suicide events: Robin Williams in 2014 (*18*) and Kate Spade and Anthony Bourdain, which occurred 3 days apart in 2018 and are here considered one event (*19*, *20*). Two observational records were used for this study. The first, total weekly calls to the National Suicide Prevention Lifeline, currently known as the 988 Suicide and Crisis Lifeline (988 Lifeline) (*21*), was used as an estimate of suicidal ideation. 988 Lifeline is a network of more than 200 round-the-clock crisis call centers that provide confidential mental health crisis and counseling services throughout the US. The second record was derived from mortality data in the National Vital Statistics System (NVSS), managed by the National Center for Health Statistics (*22*) (see the Supplementary Materials and figs. S1 and S2 for details). Our intention is to model suicide mathematically and frame understanding of suicide contagion through this dynamical lens. The two celebrity events took place during a period when suicide deaths in the US were increasing roughly 50% from 2003 to 2020 (Fig. 1A). Both celebrity suicide events were followed by a pronounced increase of reported suicide deaths (Fig. 1, B and C). Further, calls to 988 Lifeline spiked in the days and weeks following each event (Fig. 1, D and E).

Our goals for this study are to develop a model system capable of simulating suicidal behavior, to couple that system with the observations of suicidal ideation and death using an inference algorithm, and to produce reliable estimates of changes in suicide contagion following both celebrity suicide events.

## RESULTS

### Dynamic model of suicide contagion

**Fig. 2.**
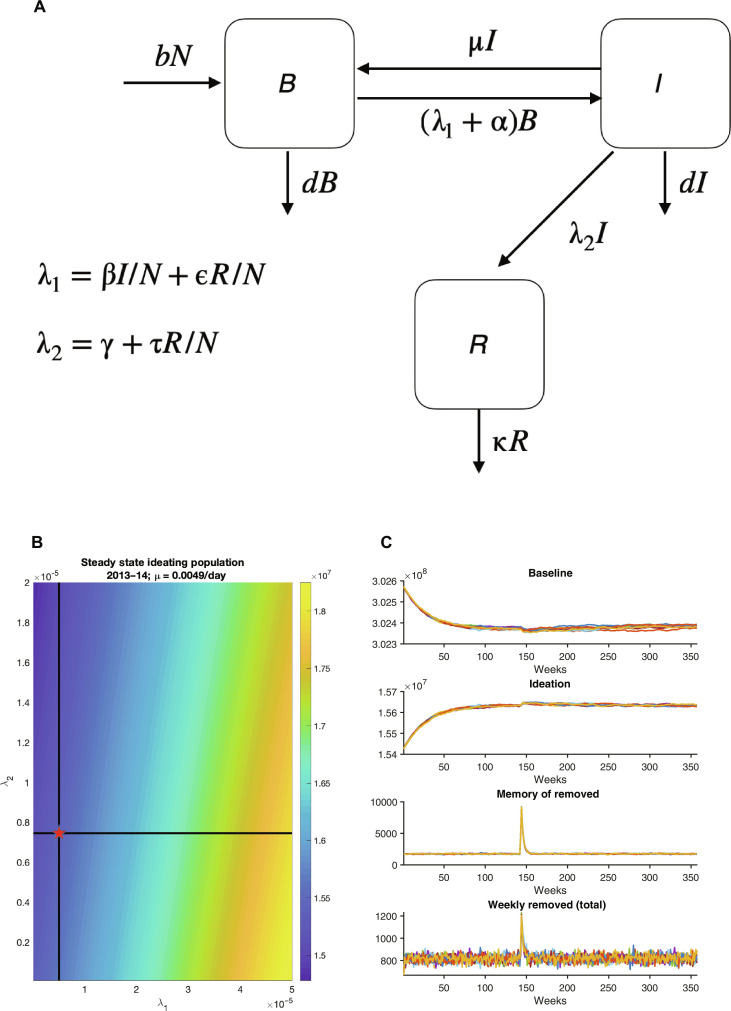
Model schematic, example steady-state solution, and free numerical simulation. (**A**) Model schematic; see the Supplementary Materials for model details and terms. (**B**) Steady-state solution of 2013–2014 US population ideating as a function of λ_1_ and λ_2_ (the forces of infection for ideation and death, respectively; see the Supplementary Materials), assuming μ = 0.0049/day. The red star indicates the steady solution for *I* = 15.423 × 10^6^ people and λ_2_ = 7.456e^−6^/day. (**C**) Ten solutions derived from the stochastic model with steady-state parameters. To illustrate the stability of the non–disease-free equilibrium, a shock displacement increasing the memory of persons who have died of suicide by 10,000 was imposed on day 1000 of each simulation. In all simulations, the integration returns to a stable equilibrium near the estimated solution.

To represent these events at US national scale, we developed a dynamic model of suicide ideation and death (Fig. 2A and see the Supplementary Materials). The model consists of three system states: (i) *B*, the baseline population currently not ideating suicide; (ii) *I*, the population ideating suicide; and (iii) *R*, the state individuals enter when they die of suicide and leave when the memory of their suicide no longer contagiously affects the living. Model dynamics are guided by parameters defining transition rates among these systems states due to individual and environmental drivers, e.g., the rate at which persons not ideating suicide, *B*, transition to suicide ideation, *I*, through noncontagious processes, as well as three parameters defining contagion transition rates among system states: (i) τ, the contagion contact rate of the population currently ideating suicide with the memory of persons who have died by suicide; (ii) β, the contagion contact rate of the population currently not ideating suicide with the population currently ideating suicide; and (iii) ϵ, the contagion contact rate of the population currently not ideating suicide with the memory of persons who have died by suicide. Our principal focus will be estimation of these three contagion parameters (τ, β, and ϵ).

### System stability

Before applying the model to actual data, we examined the stability of the model system (see the Supplementary Materials). This analysis is used to understand the behavior and properties of the model, its stability when perturbed, and to assist in differentiating baseline model behavior from celebrity suicide events. Specifically, we explored the model both analytically and numerically in order to define non–suicide-free equilibria, i.e., combinations of model state variables (*B*, *I*, and *R*) and parameters (*b*, *d*, μ, α, λ_1_, λ_2_, and κ; see Materials and Methods) at which suicide ideation and death numbers are constant and greater than zero. For both the 2014 and 2018 events, we obtained solutions using US Census Bureau national estimates of population, birth rates and death rates (*23*), 988 Lifeline call records (*21*), estimates of suicidal ideation (*24*, *25*), estimates of ideation loss rate (*26*), and the assumption that 10% of deaths by suicide derives from contagion (*27*) (see the Supplementary Materials; Fig. 2, B and C; figs. S3 to S5; and table S1). The findings indicate that stable equilibria exist for the model system even following shock displacements of conditions (Fig. 2C). Further, the model behavior in response to other extreme conditions was also examined (see figs. S6 and S7).

The findings of the stability analysis justify fixing all system parameters, except the nonlinear terms (β, ϵ, and τ), to equilibrium values (see table S1). This enables focus on estimation of the nonlinear parameters through a coupling of the model to a Bayesian inference algorithm (see the Supplementary Materials). The ability of the combined model-inference system to accurately estimate the three nonlinear parameters was assessed through synthetic testing. Specifically, using free simulations of the model with prescribed parameters and initial conditions, we generated a number of synthetic outbreaks, i.e., mock observations of weekly calls and suicide deaths, that roughly resemble 2014 and 2018 observed outcomes following the celebrity suicide deaths. We then applied the model in conjunction with the inference algorithm and mock observations (see table S2) to determine how well the model-inference system could reconstruct the prescribed nonlinear parameters and generated state conditions. Mean system estimates of the nonlinear terms (β, ϵ, and τ) were close to prescribed values (figs. S8 to S11); the estimates were more precise, as indicated by narrower 95% credible intervals, for the parameter τ, the contagion contact rate of the ideating population with the memory of persons who have died of suicide. In addition, state conditions, the numbers ideating, *I*, and the memory of persons who have died by suicide, *R*, were well estimated.

### Application to data

**Fig. 3.**
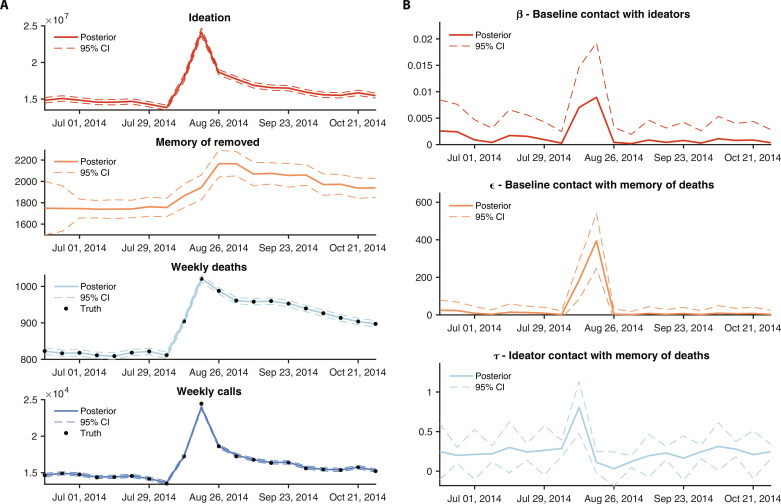
Simulation and inference for the 2014 Robin Williams suicide event. The implementation of the IF-EAKF was run 20 times, each time with a 500-member ensemble. The estimates of all 10,000 simulations for each 2-week time period were used to generate posterior estimates of the mean and distribution for the nonlinear parameters and state variables. (**A**) Weekly fitting and simulation of the model-inference system to observed outcomes. The solid lines show the mean fit; dashed lines are the 95% credible intervals. (**B**) Weekly estimates of model nonlinear parameters β, ϵ, and τ. Solid lines are the mean estimate; dashed lines are the 95% credible intervals (CIs).

We next applied the validated model-inference system to the two celebrity suicide events. Following the 2014 suicide event, a pronounced increase of all three nonlinear parameters is evident, indicating an increase in suicide contagion rates (Fig. 3). Mean estimates show a tripling of τ, the contagion contact rate of the ideating population with the memory of persons who have died by suicide, here indicating an increased likelihood of suicide death among ideators in the immediate aftermath of Robin Williams’ suicide. The effect of this increased contagion contributes to the increase of simulated deaths, which match observations. At the same time, ϵ, the contagion contact rate of the population currently not ideating suicide with the memory of persons who have died by suicide, increases more than three orders of magnitude. This shift produces a pronounced increase of the simulated population ideating suicide, which manifests in both simulated and observed call volume to 988 Lifeline. The third nonlinear parameter (β), which represents the contagion contact rate of the population currently not ideating suicide with the population currently ideating suicide, increases by an order of magnitude. This change represents an indirect response in which contact with individuals ideating suicide in the days following the 2014 suicide event is more likely to spread ideation.

**Fig. 4.**
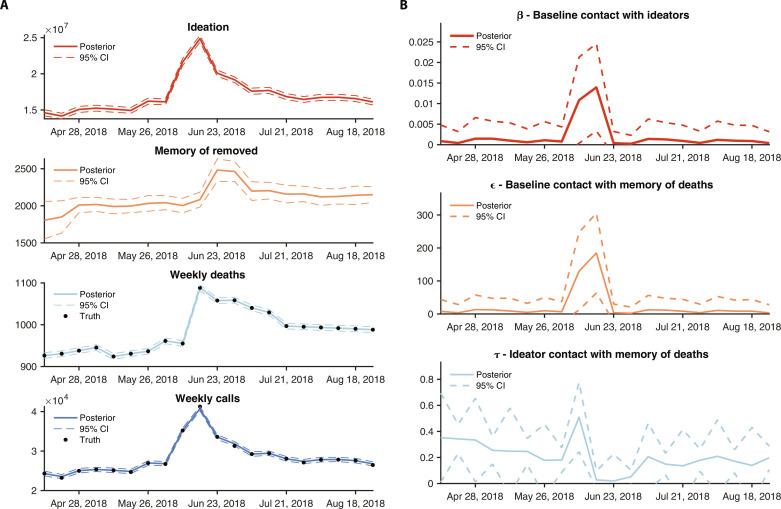
Simulation and inference for the 2018 Kate Spade/Anthony Bourdain suicide events. The implementation of the IF-EAKF was run 20 times, each time with a 500-member ensemble. The estimates of all 10,000 simulations for each 2-week time period were used to generate posterior estimates of the mean and distribution for the nonlinear parameters and state variables. (**A**) Weekly fitting and simulation of the model-inference system to observed outcomes. The solid lines show the mean fit; dashed lines are the 95% credible intervals. (**B**) Weekly estimates of model nonlinear parameters β, ϵ, and τ. Solid lines are the mean estimate; dashed lines are the 95% credible intervals.

The estimated responses following the 2018 suicide event are similar (Fig. 4); however, the magnitude of the ϵ and τ increases are roughly half. For both events the contagion response is highly transient: After 2 weeks, the contagion parameters return to pre-event levels. These results were insensitive to adjustment of the underlying fixed parameter assumptions (figs. S12 to S17). Overall, the rise in deaths following both celebrity suicide events is inferred to occur as a consequence of two changes: (i) transient increase of the contagion contact parameters (β, ϵ, and τ); and (ii) longer duration increase of *I*, the number of persons ideating suicide (Figs. 3 and 4).

## DISCUSSION

The model presented here depicts suicidal behaviors, ideation and death, as dynamical processes at population scales. Further, system coupling with an inference algorithm enables an initial quantification of contagion changes following real-world events. These estimates could be derived in real time if the observations supporting inference, 988 Lifeline call volume and NVSS suicide deaths, were available in real time. Much as for infectious disease systems, in which epidemiological characteristics such as the basic reproductive number (*R*_0_) are estimated, the results presented here do not provide a definitive absolute quantification of contagion processes but instead are also a function of the specified model form and parameter assumptions (see table S1). For this reason, more model forms need to be developed and explored, and additional quantification of contagion processes for these events and others should be conducted. Such diversity of estimates will afford comparison of modeling approaches, and examination of estimates across events will inform understanding of how high-profile suicides vary in their impact, how contagion typically and aberrantly manifests, and possible means for controlling the spread of suicidal behavior. Further, well-validated dynamical systems may ultimately be used to develop counterfactual simulation of interventions or projections of future outcomes.

Among the two celebrity suicide events, the number of excess suicide deaths was approximately double following the 2014 Robin Williams event (*11*, *28*). A similar difference is also seen in the larger magnitude increases of the contagion parameters ϵ and τ, although not the β parameter. This divergence in response may reflect differences in communication and media attention following each event (*29*, *30*), the level of population connection with the decedent, and the demographic composition of the communities most impacted by the event. It is important to note that because the model system describing suicide contagion is nonlinear, a doubling of excess suicide deaths does not necessitate a doubling of the nonlinear parameter estimates. Many combinations of changes to β, ϵ, and τ could produce a doubling of suicide deaths. For instance, increases to β and ϵ increase the numbers of people ideating suicide, the state variable *I*; if those increases are large enough, τ needs no change at all. Our system, by leveraging both death and call data, is able to disentangle and identify the most likely combination of changes to β, ϵ, and τ for a given event.

### Limitations and avenues for future research

For simplicity, we modeled the entire US population collectively; however, both events affected certain age and sex subpopulations more than others (*11*). In the future, model systems should be developed to explicitly simulate responses among these subpopulation communities and compare contagion response as a function of media and public health efforts. Further, the model developed here represents exposure to celebrity deaths by suicide; alternate dynamical model systems should be developed to depict and describe different types of exposure (*31*) and to study suicide contagion at more local scales. Network models depicting other forms of social contagion at local scales have been developed (*32*, *33*) and used in conjunction with longitudinal surveys to estimate local contagion processes such as the evolution of friendship networks, peer selection and influence, and victimization and bullying (*34*–*36*). Similar network modeling approaches might also be coupled with data on suicide ideation, attempts, and deaths to explore suicide at local scales, thus enabling quantification of these and other potential mechanisms of contagion.

Contagion responses to high-profile suicides should also be modeled for other countries, as patterns of response may differ depending on the location and celebrity. For instance, increases of excess suicide deaths following Robin Williams’ death have been documented for Canada (*37*) and Australia (*38*), but in England and Wales, no significant increase was found (*39*). Quantification of contagion parameters for these and other events across countries may shed light on how different populations are affected and, ultimately, how interventions might best be targeted. In particular, determination of the demographic and socioeconomic features associated with differences in contagion response would be beneficial.

Some of our model assumptions also require further investigation. For example, Platt *et al.* (*27*) found that clusters of suicide among youth occurred for 0.8 to 10.3% of suicide deaths. Our assumption that 10% of suicides deaths are due to contagion (see the Supplementary Materials) lies near the upper end of this range; however, clusters are not equivalent to contagion, i.e., the transmission of suicidal ideation or behavior (*5*), nor is our study population exclusively youth. Further empirical studies are needed to better quantify rates of suicide contagion across different demographic groups. In addition, fluctuations in the use of 988 Lifeline may manifest for reasons other than ideation, such as in response to media or public service campaigns. Future study of excursions in call volume is needed to better control for these factors.

Aspects of suicide contagion, in particular at local scales, are thought to manifest through changing peer influences or group norms (*5*), processes that may be relatively slow-moving; however, the model developed here finds rapid spread of suicide ideation and deaths following the suicide deaths of celebrities whose lives and work are known and likely meaningful to large portions of the population. Further work with dynamical models should help inform and refine theoretical understanding of the processes and time scales governing suicide contagion.

### Policy implications

Additional studies are needed to more fully understand the mechanisms driving suicide contagion and to inform suicide response and prevention efforts. By comparing contagion responses, as seen here in transient changes to model nonlinear parameters, across different events and populations, and by using different modeling approaches, we may improve understanding of the characteristics associated with suicide contagion spread. Such characterization may ultimately inform real-time mental health services, the targeting of suicide prevention messaging, and the directing of interventions to specific subpopulations.

The model-inference system developed here is analogous to frameworks used to generate infectious disease and weather forecasts. However, there remain substantial obstacles to real-time prediction of suicide contagion events. In particular, understanding of these events remains limited and needs to be expanded through greater documentation and quantification of suicide contagion, both locally and at population scales. Further, the real-time data critically needed to generate real-time predictions—observations of suicide ideation, attempts, and death—are typically not available until months after an index event. To enable the real-time data collection and availability needed to support operational forecasting, reporting would likely need to be automated, centrally curated, and mandated.

## MATERIALS AND METHODS

### Observations

Two observational records were used for this study. The first, total weekly calls to National Suicide Prevention Lifeline [currently known as the 988 Suicide and Crisis Lifeline (988 Lifeline)] (*21*), was used as an estimate of suicide ideation. Lifeline is a large network of more than 200 round-the-clock crisis call centers that provide confidential mental health crisis and counseling services throughout the US. Data from all connected calls during 2013–2020 were aggregated for the entire United States at weekly resolution.

The second record was derived from mortality data of the NVSS, managed by the National Center for Health Statistics (*22*). International Classification of Disease Tenth Revision (ICD-10) records from 2003–2020 were interrogated for underlying cause-of-death codes X60-X84, Y87.0, and U03 (*40*). These monthly data were aggregated for the entire US across all age groups and interpolated in time to provide an estimate of total weekly suicide deaths. See the Supplementary Materials and figs. S1 and S2 for further details.

### Model structure

A dynamical, compartmental model structure was developed to depict the transmission of suicide ideation and death at US national scales. Model equations are

$$\frac{\mathrm{dB}}{\mathrm{dt}}=\mathrm{bN}+\mu I-\alpha B-\lambda_{1}B-\mathrm{dB}$$ (1)

$$\frac{\mathrm{dI}}{\mathrm{dt}}=\lambda_{1}B+\alpha B-\mu I-\lambda_{2}I-\mathrm{dI}$$ (2)

$$\frac{\mathrm{dR}}{\mathrm{dt}}=-\kappa R+\lambda_{2}I$$ (3)

where *N* is the total population, *B* is the population currently not ideating suicide, *I* is the population ideating suicide, and *R* is the state individuals enter when they die of suicide and leave when the memory of their suicide no longer contagiously affects the living. *b* and *d* are the birth and death rates, respectively; μ is the ideation loss rate, i.e., the rate at which persons ideating suicide transition to a baseline state of not ideating suicide; α is the ideation gain rate, i.e., the rate at which persons not ideating suicide transition to suicide ideation through noncontagious processes; λ_1_is the force of infection for ideation, i.e., the rate at which persons not ideating suicide transition to suicide ideation due to contagious processes; λ_2_ is the force of infection for death, i.e., the rate at which persons ideating suicide die by suicide due to both noncontagious and contagious processes; and κ is suicide memory loss rate.

Note that *R* is neither the number of people who died by suicide nor the number of people who remember the suicide death of others. Rather, it is the average awareness of people who have died by suicide. A person who dies by suicide moves from the *I* to the *R* compartment. For a noncelebrity, the contagious impact of knowing that a person died by suicide will be acute for family, friends, and acquaintances but will wane over time (the waning rate is determined by κ, which removes people from the *R* compartment). However, averaged over the entire population of the US, given how small most individual’s social network is, the mean contagious effects on the entire US population, here represented by λ_1_ and λ_2_, will be small. This effect differs for a celebrity suicide when general population knowledge of and affinity for that celebrity is great. In that instance, the mean contagious effect for the entire US population may be much higher, possibly represented by an increase of λ_1_ and λ_2_. In effect, the suicide of a more well-known individual who many in a population identify with and value can have a much greater contagion impact. People are directly “infected” by the celebrity suicide through their affinity to the person and knowledge of the celebrity death.

The force of infection for ideation is further defined as

$$\lambda_{1}=\beta \frac{I}{N}+\epsilon \frac{R}{N}$$ (4)

where β is the contagion contact rate of the population currently not ideating suicide (*B*) with the population currently ideating suicide (*I*), and ϵ is the contagion contact rate of the population currently not ideating suicide (*B*) with the memory of persons who died by suicide. Both terms on the right-hand side (rhs) of Eq. 4 produce nonlinearities in the model (Eqs. 1 to 3), i.e., $\lambda_{1}B=\beta \frac{I}{N}B+\epsilon \frac{R}{N}B$ , and represent two contagious pathways by which individuals begin to ideate suicide.

The force of infection for death is further defined as

$$\lambda_{2}=\gamma +\tau \frac{R}{N}$$ (5)

where γ is the suicide death rate, i.e., the background rate at which persons ideating suicide die of suicide; and τ is the contagion contact rate of the population currently ideating suicide (*I*) with the memory of persons who have died by suicide. The first term on the rhs of Eq. 5 is linear and represents the noncontagious rate of suicide among ideators; the second term on the rhs of Eq. 5 is nonlinear in the model (Eqs. 1 to 3), i.e. $\lambda_{2}I=\gamma I+\tau \frac{R}{N}I$ , and represents a contagious pathway by which individuals ideating suicide are influenced to die by suicide through the awareness of others who have recently died by suicide.

The full model, Eqs. 1 to 5, is integrated stochastically with a daily time step. The dynamic model simulates the entire US population without discrimination of subgroups by geography, gender, race, or age. In this manner, it emulates simple mass balance, compartmental models of infectious disease (*6*). The daily number of suicide deaths is given by the term λ_2_*I*. Key model assumptions are provided in table S3.

### Model stability

Before numerical simulations, we performed a stability analysis to find non–suicide-free equilibria for the model system (see the Supplementary Materials). This analysis identified nontrivial model solution states at which suicide ideation and death numbers are constant and greater than zero. The analysis provided information on the behavior of the model system and informed our approach to numerical simulation and inference.

### Model-inference framework

We coupled the dynamical model with an iterated filtering (IF) approach (*41*) adapted for use with the ensemble adjustment Kalman filter (EAKF) (*42*). The combined IF-EAKF was used to assimilate the time series observations of Lifeline calls and suicide deaths and adjust the state variables and parameters of the dynamical model. Through this assimilation process, the distribution of the variables and parameters were recursively adjusted to provide estimates of both observed state conditions and unobserved state variables and parameters. See the Supplementary Materials for a fuller description of the inference process, as well as initialization and application of the IF-EAKF and synthetic testing.

### Resources

This research paper discusses suicide and self-harm. If you or someone you know is experiencing suicidal ideation or mental health distress, you can find help through a suicide-prevention or mental health crisis support line:

1. United States: Call or text “988” or go to https://988lifeline.org/.

2. United Kingdom: 116 123.

3. Others: https://en.wikipedia.org/wiki/List_of_suicide_crisis_lines.

## Supplementary Materials

### This PDF file includes:

Supplementary Materials and Methods Figs. S1 to S17 Tables S1 to S3 Legend for data S1 References
