# Supplementary material for: Quantifying suicide contagion at population scale
Source: Sci Adv. 2024 Jul 31;10(31):eadq4074. doi: 10.1126/sciadv.adq4074 (PMC11290520; doi:10.1126/sciadv.adq4074)
Supplement: Supplementary file 1 — Supplementary Materials and Methods Figs. S1 to S17 Tables S1 to S3 Legend for data S1 References [file sciadv.adq4074_sm.pdf]

Science Advances
AAAS

Supplementary Materials for

Quantifying suicide contagion at population scale

Jeffrey Shaman *et al.*

Corresponding author: Jeffrey Shaman, jls106@cumc.columbia.edu

Sci. Adv. **10**, eadq4074 (2024)
DOI: 10.1126/sciadv.adq4074

The PDF file includes:

Supplementary Materials and Methods
Figs. S1 to S17
Tables S1 to S3
Legend for data S1
References

Other Supplementary Material for this manuscript includes the following:

Data S1

Materials and Methods

1. Observations

Two observational records were employed for this study. The first, total weekly calls to the National Suicide Prevention Lifeline (currently known as the 988 Suicide and Crisis Lifeline (988 Lifeline)) (*21*), was used as an estimate of suicide ideation. 988 Lifeline is a large network of more than 200 round-the-clock crisis call centers that provide confidential mental health crisis and counseling services throughout the US. Data from all connected calls during 2013-2020 were aggregated for the entire United States at weekly resolution. Connected calls are defined as those that at a minimum were queued in a center's automated call distribution system to await answering. Repeat calls could not be identified and were thus not excluded.

The second record was derived from mortality data of the National Vital Statistics System (NVSS), managed by the National Center for Health Statistics (*22*). International Classification of Disease Tenth Revision (ICD-10) records from 2003-2020 were interrogated for underlying cause-of-death codes X60-X84, Y87.0 and U03 (*38*). These monthly data were aggregated for the entire US across all age groups to provide an estimate of total monthly suicide deaths.

Two time periods were focal for this study, each of which included high profile celebrity suicides. The first time period, 2013-14, includes the August 11, 2014 suicide of Robin Williams (RW) (*18*). The death of Mr. Williams was followed by a surge of suicide deaths in the US, particularly among males and 30-44 years olds (*11*), though also reflected in the broader population. The second time period, 2017-18, includes the June 5, 2018 and June 8, 2018 suicides of Kate Spade and Anthony Bourdain, respectively (KSAB) (*19, 20*). These suicide deaths, which occurred in quick succession, were also followed by a surge of suicide deaths in the broader US population.

Our aim in this study is to quantify suicide contagion using observations, a dynamic model of suicide ideation and death, and Bayesian inference approaches. To support this model-inference effort we de-trended daily national Lifeline calls and aggregated to weekly totals (Figure 1, main text). In addition, we removed the seasonal cycle from the monthly national NVSS suicide deaths and linearly interpolated to weekly resolution. For the period immediately preceding and following the celebrity suicide events we constrained the interpolation to ensure suicide deaths did not increase prior to the week of the event. For example, for the 3 weeks before the August 11, 2014 event, we subtracted deaths above the mean for the weeks 4-8 weeks prior to the event plus random (normal) noise. The removed deaths were then flipped chronologically and added to the weekly deaths during subsequent 3-week period. This removal and addition conserved mass and produced an estimated weekly record of deaths that only surged following celebrity suicide. A similar procedure was applied to the 2018 event (Figure 1, main text).

We note that the constraint of the interpolation of the NVSS suicide death data is justified by several factors. Firstly, there is no evidence that natural variability or exogenous factors would produce a large, anomalous increase of suicide deaths prior to either celebrity suicide event. Indeed, over the 20-year record studied here, the two celebrity events were associated with the largest observed increases in both calls and deaths by suicide (Figure S1), indicating that neither natural variability and exogenous events, including the 2008 financial bubble, have produced

anomalies of similar magnitude. Secondly, examination of hourly 988 Lifeline call data shows that increase call volume occurred after the celebrity suicide events and was coincident with and after reporting of the celebrity deaths. For example, Mr. Williams was discovered shortly before 3pm ET (12pm PT). His death broke on CNN at 7:06pm ET, but likely leaked earlier via other news outlets. Calls did not rise until after his death and grew precipitously in the hours after national reporting (Figure S2). Collectively, this information informed our expectation that suicide deaths would only increase anomalously after a celebrity suicide event.

2. Model Structure

The model developed here to simulate suicide ideation and death as a contagious process is described as:

$$\frac{dB}{dt} = bN + \mu I - \alpha B - \lambda_1 B - dB \qquad [1]$$

$$\frac{dI}{dt} = \lambda_1 B + \alpha B - \mu I - \lambda_2 I - dI \qquad [2]$$

$$\frac{dR}{dt} = -\kappa R + \lambda_2 I \qquad [3]$$

where N is the total population, B is the population currently not ideating suicide, I is the population ideating suicide, and R is the state individuals enter when they have died by suicide and leave when the memory of their suicide no longer contagiously affects the living, b and d are the birth and death rates, respectively; μ is the ideation loss rate, i.e. the rate at which persons ideating suicide transition to a baseline state of not ideating suicide; α is the ideation gain rate, i.e. the rate at which persons not ideating suicide transition to suicide ideation through non-contagious processes; $\lambda_1$is the force of infection for ideation, i.e. the rate at which persons not ideating suicide transition to suicide ideation due to contagious processes; λ_2 is the force of infection for death, i.e. the rate at which persons ideating suicide die by suicide due to both non-contagious and contagious processes; and κ is suicide memory loss rate.

Note that R is neither the number of people who died by suicide, nor the number of people who remember the suicide death of others. Rather, it is the average awareness of people who have died by suicide. A person who dies by suicide moves from the I to the R compartment. For a non-celebrity, the contagious impact of *knowing* that a person died by suicide will be acute for family, friends, and acquaintances but will wane over time (the waning rate is determined by κ, which removes people from the R compartment). However, averaged over the entire population of the US, given how small most individual's social network is, the mean contagious effects on the entire US population, here represented by λ_1 and λ_2, will be small. This effect differs for a celebrity suicide when general population knowledge of and affinity for that celebrity is great. In that instance, the mean contagious effect for the entire US population may be much higher, possibly represented by an increase of λ_1 and λ_2. In effect, the suicide of a more well-known individual who many in a population identify with and value can have a much greater contagion impact. People are directly 'infected' by the celebrity suicide through their affinity to the person and knowledge of the celebrity death.

The force of infection for ideation is further defined as:

$$\lambda_1 = \beta \frac{I}{N} + \epsilon \frac{R}{N} \qquad [4]$$

where β is the contagion contact rate of the population currently not ideating suicide (B) with the population currently ideating suicide (I), and ϵ is the contagion contact rate of the population currently not ideating suicide (B) with the memory of persons who have died by suicide. Both terms on the right-hand side (rhs) of Eq. 4 produce nonlinearities in the model (Eqs. 1-3), i.e. $\lambda_1 B = \beta \frac{I}{N} B + \epsilon \frac{R}{N} B$, and represent two contagious pathways by which individuals begin to ideate suicide.

The force of infection for death is further defined as:

$$\lambda_2 = \gamma + \tau \frac{R}{N} \qquad [5]$$

where γ is the suicide death rate, i.e. the background rate at which persons ideating suicide die by suicide; and τ is the contagion contact rate of the population currently ideating suicide (I) with the memory of persons who have died by suicide. The first term on the rhs of Eq. 5 is linear and represents the non-contagious rate of suicide among ideators; the second term on the rhs of Eq. 5 is nonlinear in the model (Eqs. 1-3), i.e. $\lambda_2 I = \gamma I + \tau \frac{R}{N} I$, and represents a contagious pathway by which individuals ideating suicide are influenced to die by suicide through the awareness of others who have recently died by suicide.

The full model, Eqs. 1-5, is integrated stochastically with a daily time step. The dynamic model simulates the entire US population without discrimination of sub-groups by geography, gender, race or age. In this manner it emulates simple mass-balance, compartmental models of infectious disease (*6*). The daily number of suicide deaths is given by the term $\lambda_2 I$.

3. Model Stability

Prior to numerical simulations, and because dynamic model forms for suicide are few (*15*), we first performed a stability analysis to find non-suicide-free equilibria for the system. This analysis aims to find non-trivial model solution states, i.e. combinations of model state variables (B, I, R) and parameters ($b, d, \mu, \alpha, \lambda_1, \lambda_2, \kappa$) at which suicide ideation and death numbers are constant and greater than zero. Such analyses provide useful information on the behavior of the model system and inform the approach to numerical simulation.

3.1. Equilibria Solution

Model equilibria are found by setting Eqs. 1 and 2 equal to zero, such that

$$bN + \mu I = (\lambda_1 + \alpha + d)B$$

$$(\alpha + \lambda_1)B = (\mu + d + \lambda_2)I$$

Assuming the birth rate and death rate are equal, $b = d$, and solving for I yields:

$$I = \frac{N}{1+\frac{(\mu+d+\lambda_2)}{(\alpha+\lambda_1)}+\frac{\lambda_2}{d}} \qquad [6]$$

Equation 6 indicates that increasing λ_1 increases I at equilibrium and increasing λ_2 decreases I at equilibrium.

We next look to find equilibria solution using realistic parameters. The population of study is the US. During 2013-2014, the period of the Robin Williams suicide, the US population was approximately 318 million. We impose equal birth and death rates of 1% per year, or $b = d = 2.74 \times 10^{-5}/day$. For the equilibrium solution, we assume that $\alpha B \approx \mu I$, i.e. that the linear ideation gain and loss terms are approximately equivalent.

We derived an estimate of the percentage of the US population ideating from the National Center for Health Statistics (NCHS) 2013-2014 National Health and Nutrition Examination Survey (NHANES) Mental Health – Depression Screener (DPQ_H) survey (*24, 25*). Specifically, we used survey question DPQ090 – 'Over the last 2 weeks how often have you been bothered by thoughts you would be better off dead?' Of 5390 respondents, 122 answered 'several days', 31 answered 'more than half the days', and 32 answered 'every day'. These 3 responses combined constitute 3.43% of respondents. When weighted for the gender and age biases of the respondents relative to the US population, 4.85% (standard error 0.52%) of the US population is estimated to have been ideating suicide at the time of this survey. Using this estimate implies $I = 0.0485N$. Given $N = B + I \Rightarrow B = 0.9515N$.

Estimates of ideation gain and loss are limited; however, one study (*26*) conducted baseline and 12-month follow-up surveys on suicidal ideation among nearly 1,000 Hong Kong residents ages 20-59. Among 66 individuals found to be ideating suicide at the time of the baseline survey, 55 were no longer ideating one year later. This finding translates to an exponential ideation loss rate of $\mu = 0.0049/day$. Given this estimate and our equilibrium assumption, the ideation gain rate is:

$$\alpha \approx \mu \frac{I}{B} = 0.00025/day$$

We apply these numbers to Eq. 6, i.e.

$$I = \frac{318{,}000{,}000\ persons}{1+\frac{\left(\frac{0.0049}{day}+\frac{2.74\times10^{-5}}{day}+\lambda_2\right)}{\left(\frac{0.00025}{day}+\lambda_1\right)}+\frac{\lambda_2}{2.74\times10^{-5}/day}} [7]$$

Figure 2, main text, shows numerical solutions of Eq. 7 for a range of λ_1 and λ_2. During 2013-2014 approximately 42,000 people died by suicide each year in the US, or $\sim 115/day$. $I = 0.0485N = 15.423$ million people, indicating a force of infection of suicide death of $\lambda_2 = 7.456 \times 10^{-6}/day$. Given this additional information, the non-suicide-free equilibrium solution for this system thus must satisfy:

$$15{,}423{,}000\,persons = \frac{318{,}000{,}000\,persons}{1 + \frac{\left(\frac{0.0049}{day} + \frac{2.74 \times 10^{-5}}{day} + \frac{7.456 \times 10^{-6}}{day}\right)}{\left(\frac{0.00025}{day} + \lambda_1\right)} + \frac{7.456 \times 10^{-6}/day}{2.74 \times 10^{-5}/day}}$$

This corresponds to $\lambda_1 = 5.078 \times 10^{-6}/day$ (Figure 2, main text). Similar calculations are used to derive steady-state estimates assuming $\mu = 0.01$ (Figure S3) and for the 2017-2018 time period (Figures S4 and S5).

3.2. Numerical Solution

We next want to impose the above conditions in the model system (Eqs. 1-5) and validate that these conditions produce an equilibrium solution. To do so, we first need to specify the components of Eqs. 3-5. Suicide memory loss, κ, is a quantity for which there is little information. It represents the rate at which the contagious effects of a suicide decay. Here, we impose a rate of $\kappa = 0.0667/day$ or an e-folding period of 15 days. With this assumption, we can use Eq. 3 to estimate the equilibrium state for R:

$$0.0667R = \frac{7.456 \times 10^{-6}}{day} 1.5423 \times 10^{7} persons \Rightarrow R \approx 1724\,persons$$

For Eq. 4, we have $\lambda_1 = \beta \frac{I}{N} + \epsilon \frac{R}{N} = 5.078 \times 10^{-6}/day$. If we assume that only 10% of this force of infection for ideation is derived from suicide deaths, then $\epsilon \frac{R}{N} = 5.078 \times \frac{10^{-7}}{day} \Rightarrow \epsilon = 0.0937/day$. The other 90% of ideation contagion is thus derived from ideators such that $\beta \frac{I}{N} = 4.57 \times \frac{10^{-6}}{day} \Rightarrow \beta = 0.00009423/day$.

For Eq. 5, we assume that only 10% of suicide deaths are due to contagion, i.e. $\gamma = 6.71 \times 10^{-6}/day$ and $\tau \frac{R}{N} = 7.456 \times \frac{10^{-7}}{day} \Rightarrow \tau = 0.1375/day$.

The call rate, ρ, was calculated directly from typical daily 988 Lifeline call rates and estimated numbers of ideators. For example, during 2013 there were approximately 2000 calls to 988 Lifeline per day and there were 15.423 million estimated ideators on any given day, such that

$$\rho = \frac{2000 \frac{\text{calls}}{\text{day}}}{15.423 \times 10^{6}\ \text{ideators}} = 1.297 \times 10^{-4}\ {}^{\text{calls/day}}\!/_{\text{ideator}}$$

We next imposed the parameters derived from these assumptions and the equilibria solution (Table S1) in numerical simulations with the full model to validate that a stable numerical equilibrium exists. Note that the value for the parameter d, the death rate, was lowered slightly to compensate for additional deaths due to suicide. Figure 2, main text, presents 10 solutions derived from the stochastic model with these parameters. A shock displacement increasing the memory of persons who died by suicide by 10,000 was imposed on Day 1,000 of each

simulation. In all simulations, the integration converges to a stable equilibrium near the estimated solution. Similar solutions are found for other equilibria solutions (Figures S1-S3).

4. Extreme Condition Tests

To further test the validity and sensitivity of the model, we ran additional free simulations, using the initial conditions and parameters from Figure 2, with the following changes:

i. Initial conditions of $I_0 = 0$ and $R_0 = 0$, such that no one is initially ideating suicide and there is no memory of deaths by suicide; however, α, the ideation gain rate remains positive (Figure S6, left). With these conditions, the system ramps up to equilibrium, as expected, over a period of approximately 2 years.

ii. Initial conditions of $I_0 = 0$, $R_0 = 0$, and $\alpha = \beta = \epsilon = 0/day$. With these parameter changes the model, as expected, produces no suicide ideation or deaths, even with a shock displacement of R (Figure S6, right). Note the population slowly increases as the birth rate slightly exceeds the death rate.

iii. Initial conditions of $I_0 = 3.15 \times 10^8$ and $R_0 = 50{,}000$, such that 99% of the population is initially ideating suicide and there is a very large number of people whose memory contagiously affects the living (Figure S7, left). As expected with these initial conditions, an outbreak of suicide deaths ensues. Approximately 79 million people die during the first year. The outbreak is self-limited due to the continual movement of ideators (I) to baseline (B), due to the parameter μ, the ideation loss rate. The states (B, I, R) move to new equilibria with a markedly reduced total population.

iv. Initial conditions of $I_0 = 3.15 \times 10^8$, $R_0 = 50{,}000$, and $\beta = \epsilon = \tau = 0/day$. As expected, with the nonlinear parameters set to zero, no outbreak is possible despite the extreme initial conditions, and the system decays to equilibrium (Figure S7, right).

5. Model-Inference Framework

Our principal objective is to couple the full model, Eqs. 1-5, with time series observations and data assimilation approaches. This model-inference framework will then be tested for identifiability (Section 7, below) and applied to actual observations (Section 1, above).

We infer model epidemiological parameters using an iterated filtering (IF) approach (*41*). The original IF framework, built around a particle filter, infers the maximum likelihood estimates of parameters in epidemic models and has been successfully applied to infectious diseases such as cholera (*43*) and measles (*44*). In these applications, the IF framework consists of an ensemble of system states, which represent the distribution of parameters and variables. The state variables are dynamically evolved per model equations, and the parameters are estimated using a particle filter. Multiple iterations, or passes through the complete time series of observations, are employed during which the ensemble variance is gradually tuned down. Through this iterative

process, the distribution of parameters is recursively adjusted, per observations and converges to a maximum likelihood solution.

The IF approach has also been adapted for use with the ensemble adjustment Kalman filter (EAKF) (*42*), an efficient algorithm well-suited for use with high dimensional systems and applied to disease such as influenza (*45*) and SARS-CoV-2 (*46-48*). Particle filters require a large number of particles (*49*), whereas the EAKF can generate similar results with only a few hundred ensemble members.

To represent the state-space distribution, the EAKF maintains an ensemble of system state vectors acting as samples from the distribution. The EAKF assumes that both the prior distribution and likelihood are Gaussian, and thus can be fully characterized by their first two moments (mean and variance). The update scheme for ensemble members is computed using Bayes rule (posterior ∝ prior × likelihood) via the convolution of these two Gaussian distributions. Specifically, the posterior of the ith ensemble member is updated through

$$o_{t,post}^{i} = \frac{\sigma_{t,obs}^{2}}{\sigma_{t,obs}^{2} + \sigma_{t,prior}^{2}} \bar{o}_{t,prior} + \frac{\sigma_{t,prior}^{2}}{\sigma_{t,obs}^{2} + \sigma_{t,prior}^{2}} o_{t} + \sqrt{\frac{\sigma_{t,obs}^{2}}{\sigma_{t,obs}^{2} + \sigma_{t,prior}^{2}}} \left(o_{t,prior}^{i} - \bar{o}_{t,prior}\right).$$

Here $o_{t,post}^{i}$ and $o_{t,prior}^{i}$ are the posterior and prior of the observed variable for the ith ensemble member at time t; $\bar{o}_{t,prior}$ is the mean of the prior observed variable; $\sigma_{t,obs}^{2}$ and $\sigma_{t,prior}^{2}$ are the variances of the observation and the prior observed variable; and o_t is an observation at time t. Unobserved variables and parameters are updated through their covariability with the observed variable, which can be computed directly from the ensemble. In particular, the ith ensemble member of unobserved variable or parameter x^i is updated by

$$x_{t,post}^{i} = x_{t,prior}^{i} + \frac{\sigma\left(\left\{x_{t,prior}\right\}_{n}, \left\{o_{t,prior}\right\}_{n}\right)}{\sigma_{t,prior}^{2}} \left(o_{t,post}^{i} - o_{t,prior}^{i}\right).$$

Here $x_{t,post}^{i}$ and $x_{t,prior}^{i}$ are the posterior and prior of the unobserved variable or parameter for the ith ensemble member at time t; and $\sigma\left(\left\{x_{t,prior}\right\}_{n}, \left\{o_{t,prior}\right\}_{n}\right)$ is the covariance between the prior of the unobserved variable or parameter $\left\{x_{t,prior}\right\}_{n}$ and the prior of the observed variable $\left\{o_{t,prior}\right\}_{n}$ at time t. In the EAKF, variables and parameters are updated deterministically so that the higher moments of the prior distribution are preserved in the posterior.

The EAKF was used to assimilate the two observational time series—de-trended weekly total 988 Lifeline calls and the weekly interpolation of monthly NVSS suicide deaths with the seasonal cycle removed (see Section 1, SI, above)—into the full model. We assumed a heuristic observation error variance, $\sigma_{t,s}^{2}$, for both measures based on variance of weekly time series observations for the time period directly preceding and following the celebrity suicide events, $o_{t,s}$, defined as the variance of each time series divided by 100, i.e.

$$\sigma_{t,s}^{2} = var(o_{t,s})/100$$

where the subscript s indicates the observation source: 988 Lifeline or NVSS.

Our inference focus is estimation of the contagion parameters β, ϵ, and τ. Rather than generate a single estimate for each of these three parameters over the entire length of the observational times series, the IF-EAKF framework was instead applied sequentially to generate parameter estimates for two-week blocks of time. This approach allowed the parameter estimates to change through time with each two-week period and was adopted with the recognition that suicide contagion processes are protean and may change rapidly. Initialization, application and validation of the IF-EAKF system, as applied in two-week blocks, are described more fully in Sections 6 and 7 of the SI. The IF-EAKF algorithm proceeds per the pseudo-code shown in Algorithm 1.

Algorithm 1. IF-EAKF – Applied over Successive Two-Week Time Periods

---

Input: The model $\mathcal{M}$ (Eqs. 1-5), weekly national observational estimates $\{o_{t,s}\}$ from 988 Lifeline and NVSS, the observational error variance (OEV) $\{\sigma_{t,s}^2\}$, the initial system mean state $\bar{x}_0$, a discount factor $a \in (0,1)$, the number of iterations L, and the number of two-week time periods W.

Generate an ensemble of initial system state with n members randomly selected from uniform distributions: $\{\hat{x}_0^0\}_n \sim U(\bar{x}_0 - \varepsilon, \bar{x}_0 + \varepsilon)$.

for $w = 1$ to W **do**

  for $l = 1$ to L **do**

    for $t = 14w - 13$ to $14w$ **do**

      Run model $\mathcal{M}$ from the initial system state or posterior $\{\hat{x}_l^{t-1}\}_n$ obtained from the previous update from $t = 14w - 14$ to $t = 14w - 7$ and return the ensemble of states, $\mathcal{M}(\{\hat{x}_l^t\}_n)$.

      Update the prior distribution $\{x_{l,s}^t\}_n \equiv \mathcal{M}(\{\hat{x}_l^t\}_n)$ to posterior $\{\hat{x}_{l,s}^t\}_n$ using the EAKF: $\{\hat{x}_{l,s}^t\}_n = EAKF(\{x_{l,s}^t\}_n, \{o_{t,s}\}, \{\sigma_{t,s}^2\})$.

      Run model $\mathcal{M}$ from the posterior $\{\hat{x}_l^{t-1}\}_n$ obtained from the previous update from $t = 14w - 7$ to $t = 14w$ and return the ensemble of weekly states, $\mathcal{M}(\{\hat{x}_l^t\}_n)$.

      Update the prior distribution $\{x_{l,s}^t\}_n \equiv \mathcal{M}(\{\hat{x}_l^t\}_n)$ to posterior $\{\hat{x}_{l,s}^t\}_n$ using the EAKF: $\{\hat{x}_{l,s}^t\}_n = EAKF(\{x_{l,s}^t\}_n, \{o_{t,s}\}, \{\sigma_{t,s}^2\})$.

    end for

    Generate an ensemble of system state variables for the next iteration with n members derived from normal distributions: $\{\hat{x}_{l+1}^w\}_n \sim \mathcal{N}\left(\bar{x}_l, \Sigma^{a^l}\right)$.

  end for

  Generate an ensemble of system state variables for the next two-week time period with n members randomly selected from normal distributions: $\{\hat{x}_0^{w+1}\}_n \sim \mathcal{N}(\bar{x}_L^w, \Sigma)$.

  Generate an ensemble of system parameters for the next two-week time period with n members randomly selected from uniform distributions: $\{\hat{x}_0^{w+1}\}_n \sim U(\bar{x}_L^w - \varepsilon, \bar{x}_L^w + \varepsilon)$.

end for

Output: $\bar{x}_L^w$: estimates of the system state variables and parameters for each two-week period, w.

---

6. Initialization and Application of the IF-EAKF framework.

In application, we used the steady state solution to define the model linear parameters $(b, d, \mu, \alpha, \gamma, \kappa, \rho)$ and applied the IF-EAKF framework to estimate the state variables (B, I, R) and nonlinear parameters (β, ϵ, τ). The state variables vary continuously as the model, Eqs. 1-5, is integrated, and are adjusted on a weekly basis with each new set of observations using the IF-EAKF framework. The nonlinear parameters are similarly adjusted on a weekly basis and estimated over successive two-week blocks. Inference was aligned so that the RW and KSAB suicides occurred during the first week of their respective two-week blocks. For the KSAB event, the system was run with Sunday as the first day of each two-week block. Mr. Williams' suicide occurred on a Monday and was associated with a very large increase of Lifeline calls in the following days (Figure 1); to support system stability, inference of the RW event was run with Tuesday as the first day of each two-week block. Initial prior ranges for the state variables and nonlinear parameters are listed in Table S2.

During simulation and assimilation of data, multiplicative inflation (*50*) was used to counter filter divergence and space re-probing (*51*) was used to force broad exploration of the solution space. As indicated in Algorithm 1, the IF-EAKF was applied successively to each two-week block. The final posterior state variable estimates of the prior iterative loop ($\bar{x}_L^w$) were used to initialize (I, R) for the next two-week time period as $\{\hat{I}_0^{w+1}\}_n \sim \mathcal{N}(\bar{I}_L^w, 5 \times 10^6)$ and $\{\hat{R}_0^{w+1}\}_n \sim \mathcal{N}(\bar{R}_L^w, 250)$ for each ensemble member n. L was set to 10 iterations. The third state variable, B, was calculated as the remaining population, i.e. $\{\hat{B}_0^{w+1}\}_n = N - \{\hat{I}_0^{w+1}\}_n - \{\hat{R}_0^{w+1}\}_n$. The nonlinear parameters employed the same initial prior range for each two-week time period (Table S2).

For each iteration of the IF-EAKF, the standard deviation of the estimated state variables was shrunk by a factor $a \in (0,1)$. The discount factor, a, typically ranges between 0.9 and 0.99, and was here set to $a = 0.95$. If a is too small, the algorithm may 'quench' too fast and fail to find the MLE; if it is too close to 1, the algorithm may not converge in a reasonable time interval. The number of iterations required for this convergence was determined by inspecting the evolution of posterior parameter distributions. In our implementation, we used $n = 500$ ensemble members, a shrinking parameter $a = 0.95$ and an iteration number $L = 10$.

This implementation of the IF-EAKF was run 20 times for each event. The estimates of all 10,000 simulations for each two-week time period were used to generate posterior estimates of the mean and distribution for the non-linear parameters. Key model assumptions are provided in Table S3.

7. Synthetic Testing and System Identifiability

We conducted synthetic tests to demonstrate the identifiability of the nonlinear parameters and state variables. Specifically, we generated synthetic outbreaks using free simulations of the model (Eqs. 1-5) with specified parameters and initial state variable conditions. These stochastic simulations produced time series of mock observations of weekly calls and suicide deaths. We then applied the model in conjunction with the IF-EAKF algorithm and mock observations to

determine how well the model-inference system could estimate the nonlinear parameters and state variables.

We generated a synthetic outbreak using the 2013-14 linear parameters $(b, d, \mu, \alpha, \gamma, \kappa, \rho)$ set to steady state values (see Table S1); here $\mu = 0.0049/day$ and $\alpha = 0.00025/day$. The initial conditions were: $N = 318{,}000{,}000\ persons$; $I(0) = 15{,}600{,}000\ persons$; $R(0) = 1724\ persons$; and $B = N - I(0) - R(0)\ persons$. A time series of values varying every 2 weeks for the 3 nonlinear parameters (β, ϵ, τ) was imposed in order to roughly replicate the excess calls and deaths observed following the 2014 and 2018 celebrity suicide events (see dashed black lines in Figure S8). The IF-EAKF algorithm is able to estimate the nonlinear, contagion parameters. The mock observations, or 'truths', are generally well bounded by the 95% posterior credible intervals, and the mean estimate is near the truth. The state variables are also well estimated, particularly the observed quantities—weekly calls and deaths.

To further validate the inference approach, we also tested the system on other synthetic outbreaks, including those generated with $\mu = 0.01/day$ and $\alpha = 0.00051/day$ (Figure S9), alternate changes to the nonlinear parameters (Figure S10), or higher observational error variance (Figure S11). Overall, the synthetic tests indicate that the nonlinear parameters—our main objective for quantifying suicide contagion—are well estimated.

8. Application to Celebrity Suicide Events

We used the weekly national estimates of 988 Lifeline calls and NVSS deaths. The linear parameters $(b, d, \mu, \alpha, \gamma, \kappa, \rho)$ were set to steady state values (Table S1). As a sensitivity analysis, two combinations of μ and α were utilized (Table S1). Initial conditions for the state variables and nonlinear parameters are listed in Table S2. Similar to the synthetic tests (Section 7), the linear parameters were fixed and only the nonlinear parameters and state variables were estimated using the IF-EAKF. The results with $\mu = 0.0049/day$ and $\alpha = 0.00025/day$ are presented in the main text (Figures 3 and 4); sensitivity tests with $\mu = 0.01/day$ and $\alpha = 0.00051/day$ are shown in Figures S12 and S13; with the assumption that 7% of suicide deaths are due to contagion ($\gamma = 6.93 \times 10^{-6}/day$, Figure S14); with the assumption that 4% of suicide deaths are due to contagion ($\gamma = 7.16 \times 10^{-6}/day$, Figure S15); and with $\kappa = 0.0333/day$ or $\kappa = 0.1/day\ i$ (Figures S16 and S17). Findings are similar for these sensitivity analyses, though the peak estimate of the parameter ϵ increases with $\kappa = 0.1/day$.

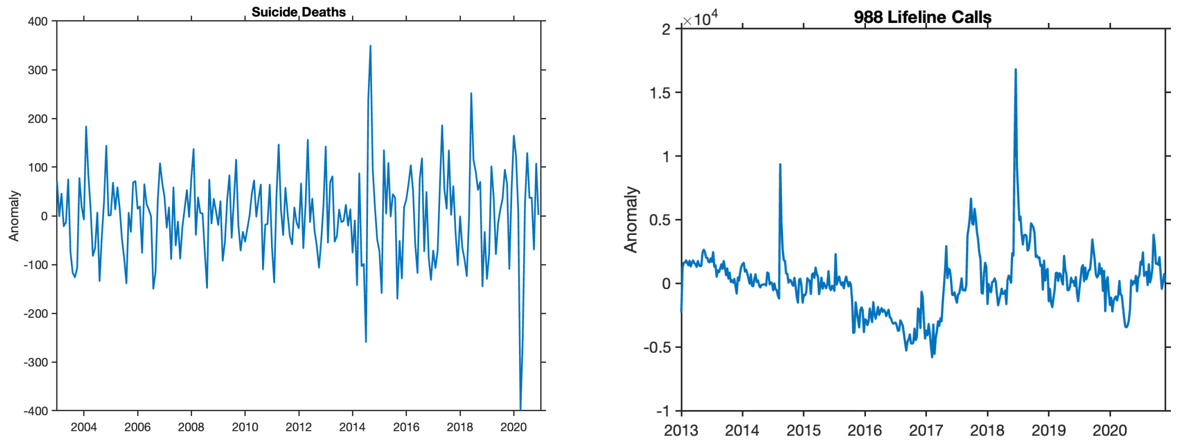

Figure S1. Suicide death and 988 Lifeline call anomalies. Left) Anomalous monthly US National NVSS suicide deaths, 2003-2020, detrended and with the seasonal cycle removed. The 2 largest positive anomalies coincide with the months of the 2 celebrity suicide events (August 11, 2014 and June 5 and 8, 2018). Note the still larger negative anomaly concurrent with pandemic lockdown. Right) Anomalous weekly 988 Lifeline calls, 2013-2020, detrended and with the seasonal cycle removed. The 2 largest positive anomalies coincide with the 2 celebrity suicide events.

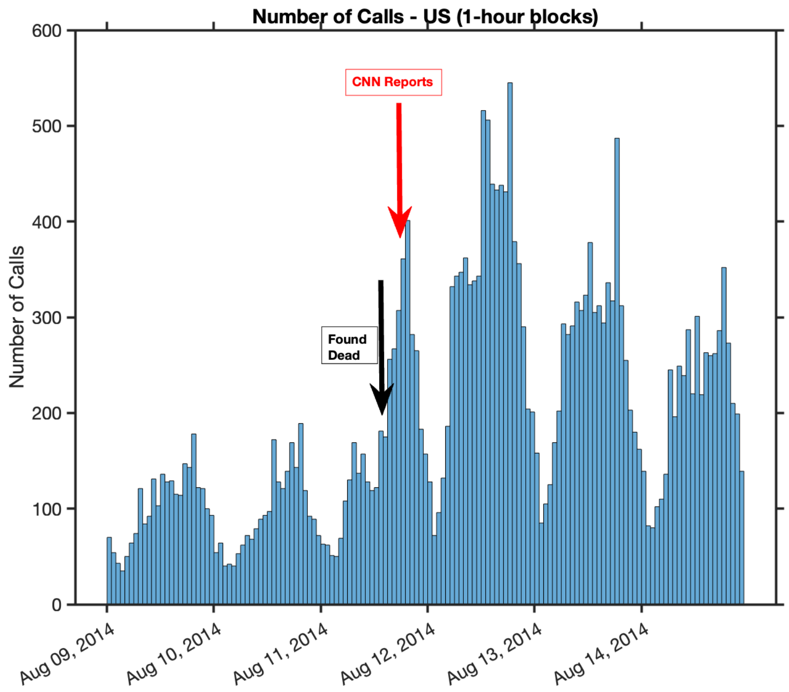

Figure S2. Hourly calls to 988 Lifeline during August 2014. The hour of the discovery of Mr. Williams' death and the reporting of the story on CNN are indicated by arrows.

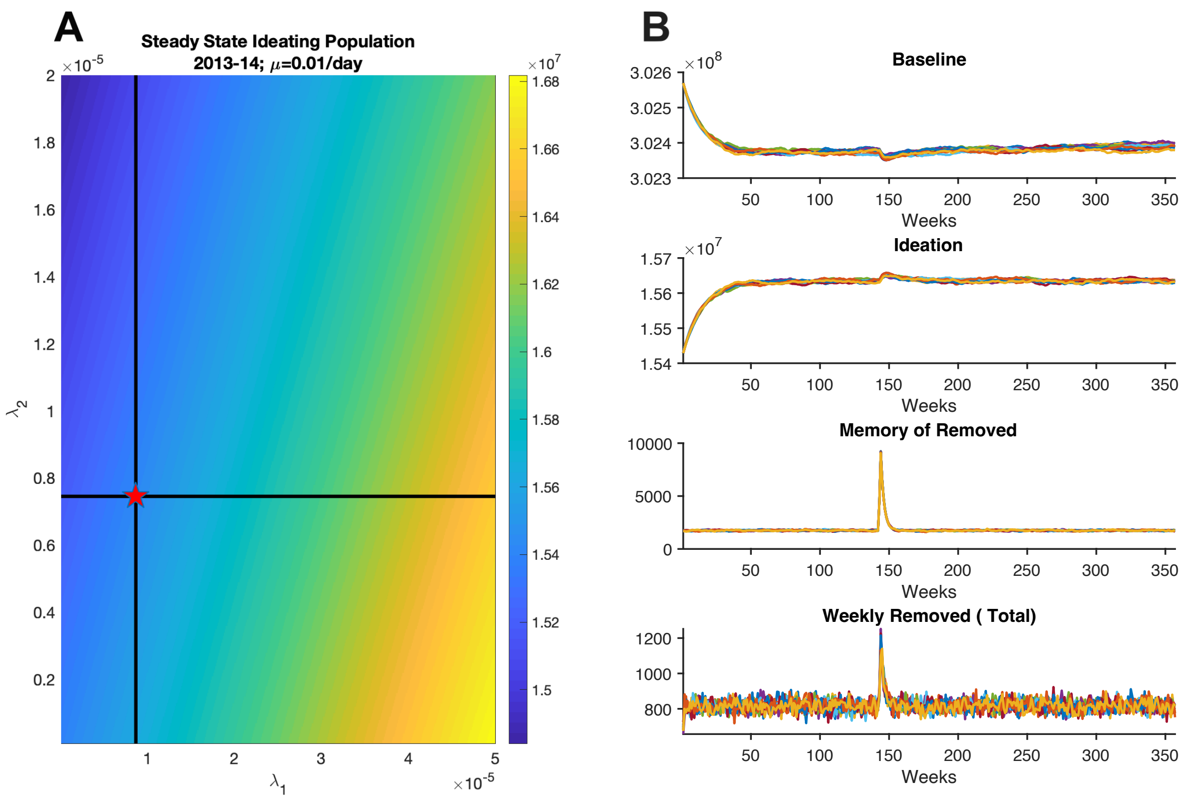

Figure S3. Steady state solution and numerical simulation. A) Steady state solution of 2013-2014 US population ideating as a function of λ_1 and λ_2, assuming $\mu = 0.01/day$. The red star indicates the steady solution for $I = 15.423 \times 10^6$ people and $\lambda_2 = 7.456e^{-6}/day$. B) Ten solutions derived from the stochastic model with steady-state parameters. To illustrate the stability of the non-disease-free equilibrium, a shock displacement increasing the memory of persons who died of suicide by 10,000 was imposed on Day 1,000 of each simulation. In all simulations, the integration returns to a stable equilibrium near the estimated solution.

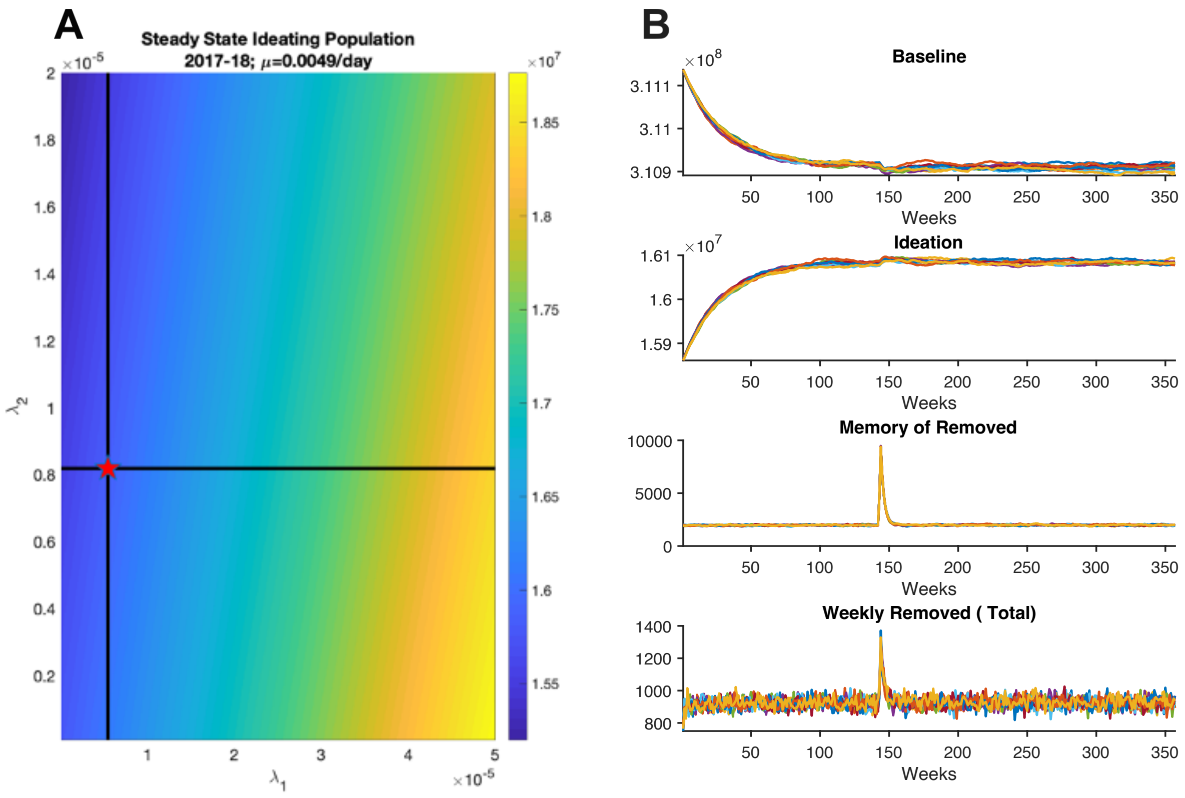

Figure S4. Steady state solution and numerical simulation. As in Figure S3 but for 2017-2018 and $\mu = 0.0049/day$. See Table S1 for parameter values.

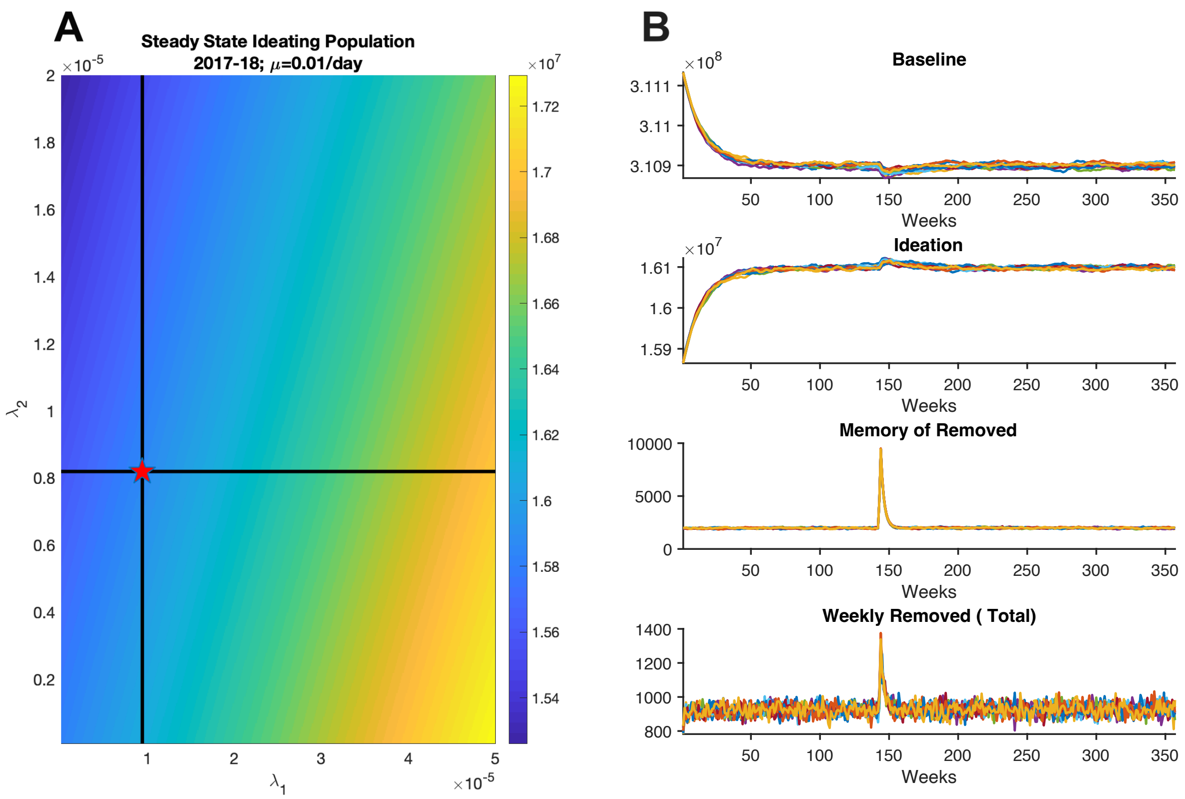

Figure S5. Steady state solution and numerical simulation. As in Figure S4 but assuming $\mu = 0.01/day$ and thus $\alpha = 0.00051/day$.

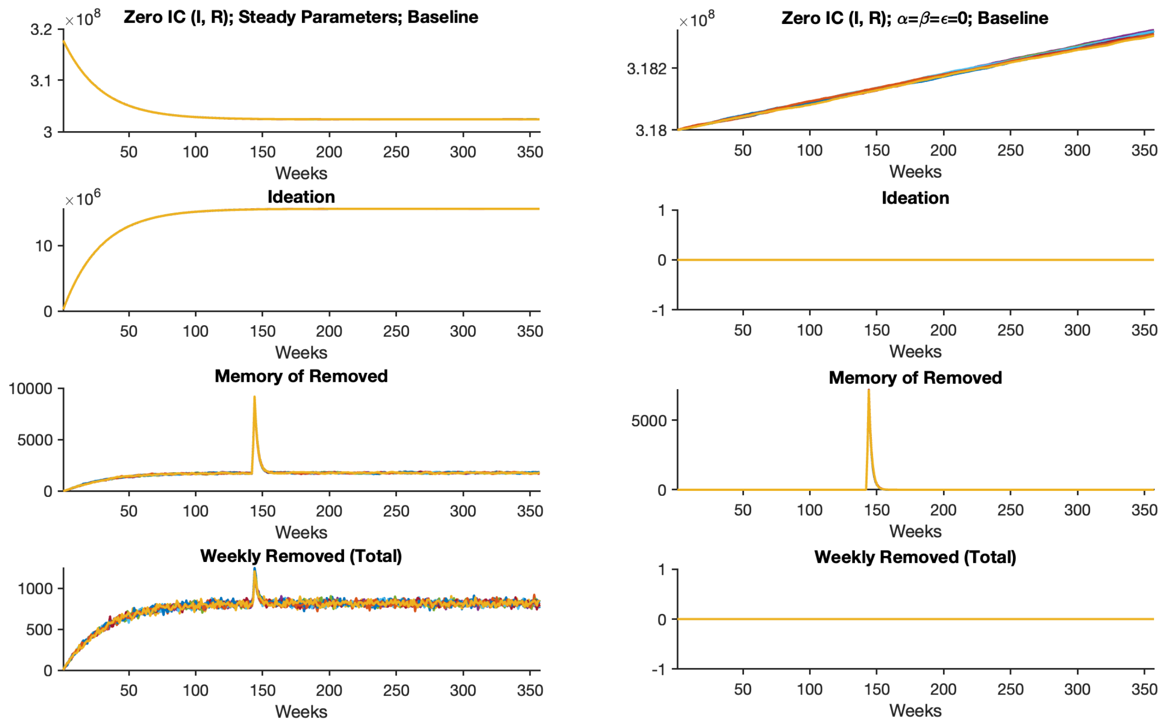

Figure S6. **Extreme Condition Tests.** Left) Ten free simulations with initial conditions of $I_0 = 0$ $R_0 = 0$. Right) Free simulation with initial conditions of $I_0 = 0$, $R_0 = 0$, and $\alpha = \beta = \epsilon = 0/day$. A shock displacement increasing the memory of persons who died of suicide by 10,000 was imposed on Day 1,000 of all simulations.

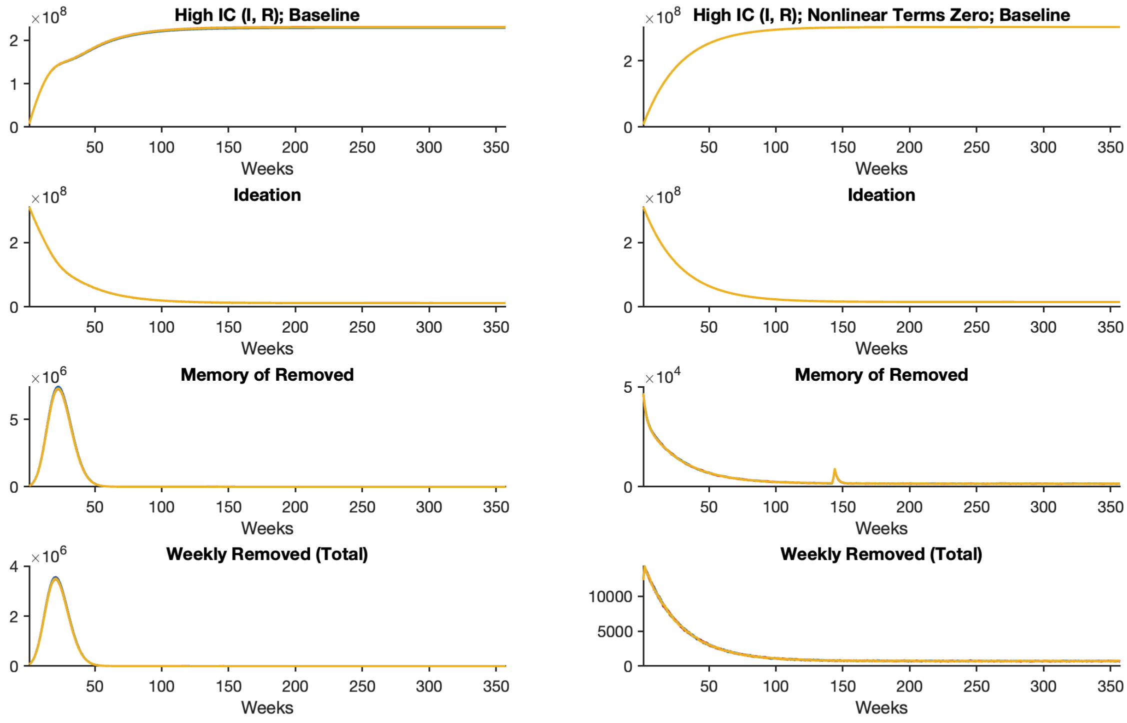

Figure S7. **Extreme Condition Tests.** Left) Ten free simulations with initial conditions of $I_0 = 3.15 \times 10^8$ and $R_0 = 50{,}000$. Right) Free simulation with initial conditions of $I_0 = 3.15 \times 10^8$, $R_0 = 50{,}000$, and $\beta = \epsilon = \tau = 0/day$. A shock displacement increasing the memory of persons who died of suicide by 10,000 was imposed on Day 1,000 of all simulations.

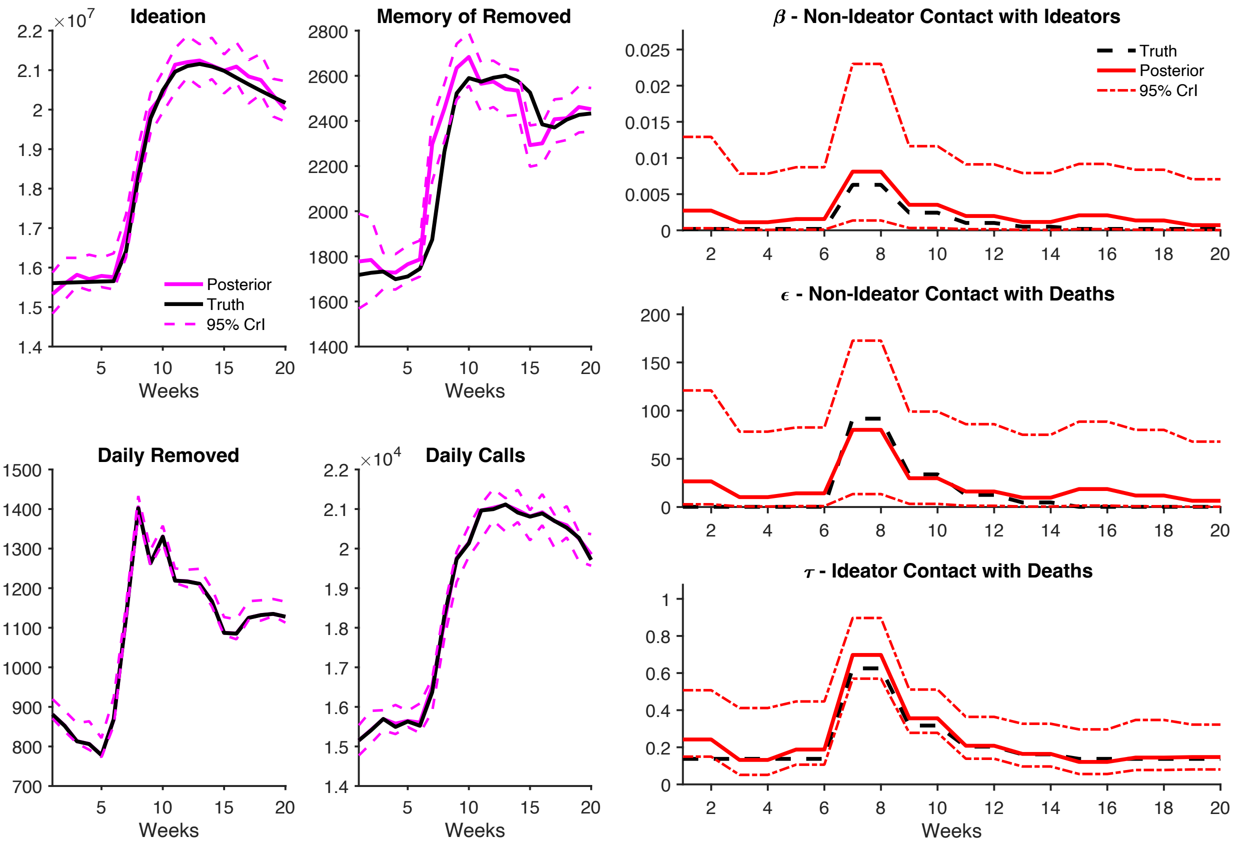

Figure S8. Synthetic test of the model-inference system. 2013-14 steady state conditions were imposed for the linear parameters (see Table S1); here $\mu = 0.0049/day$ and $\alpha = 0.00025/day$. A synthetic outbreak of suicide was generated by increasing the nonlinear parameters β, ϵ, τ. The magnitudes of increase were chosen to roughly replicate the excess calls and deaths observed following some celebrity suicide events. Using the synthetic observations, the implementation of the IF-EAKF was run 20 times, each time with a 500-member ensemble. The estimates of all 10,000 simulations for each two-week time period were used to generate posterior estimates of the mean and distribution for the non-linear parameters and state variables. Left) Estimation of state variables; the dashed black line is the truth; the solid magenta line is the mean model-inference estimate; the dash-dot magenta lines are the estimated 95% credible intervals. Right) Estimation of the nonlinear parameters; the dashed black line is the truth; the solid red line is the mean model-inference estimate; the dash-dot red lines are the estimated 95% credible intervals.

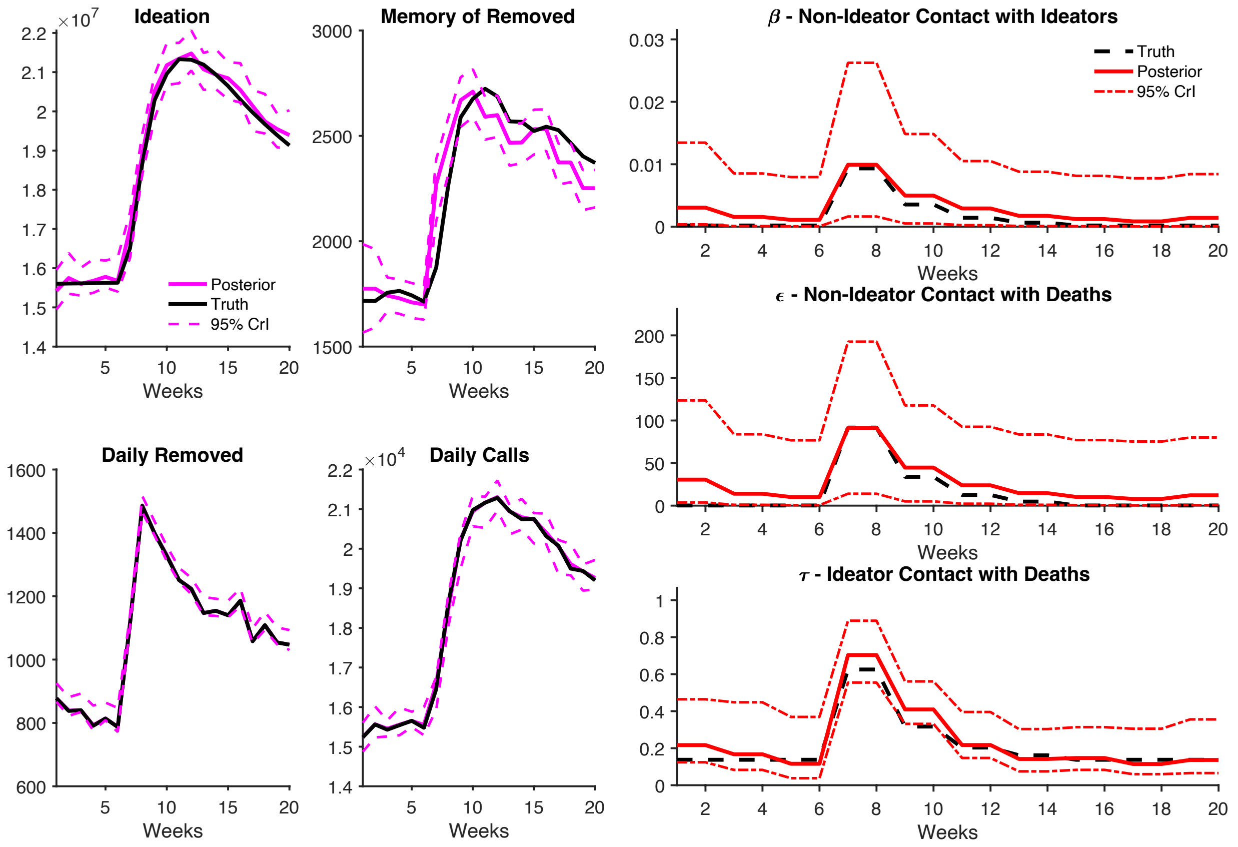

Figure S9. Synthetic test of the model-inference system. As for Figure S8, but with $\mu = 0.01/day$ and $\alpha = 0.00051/day$.

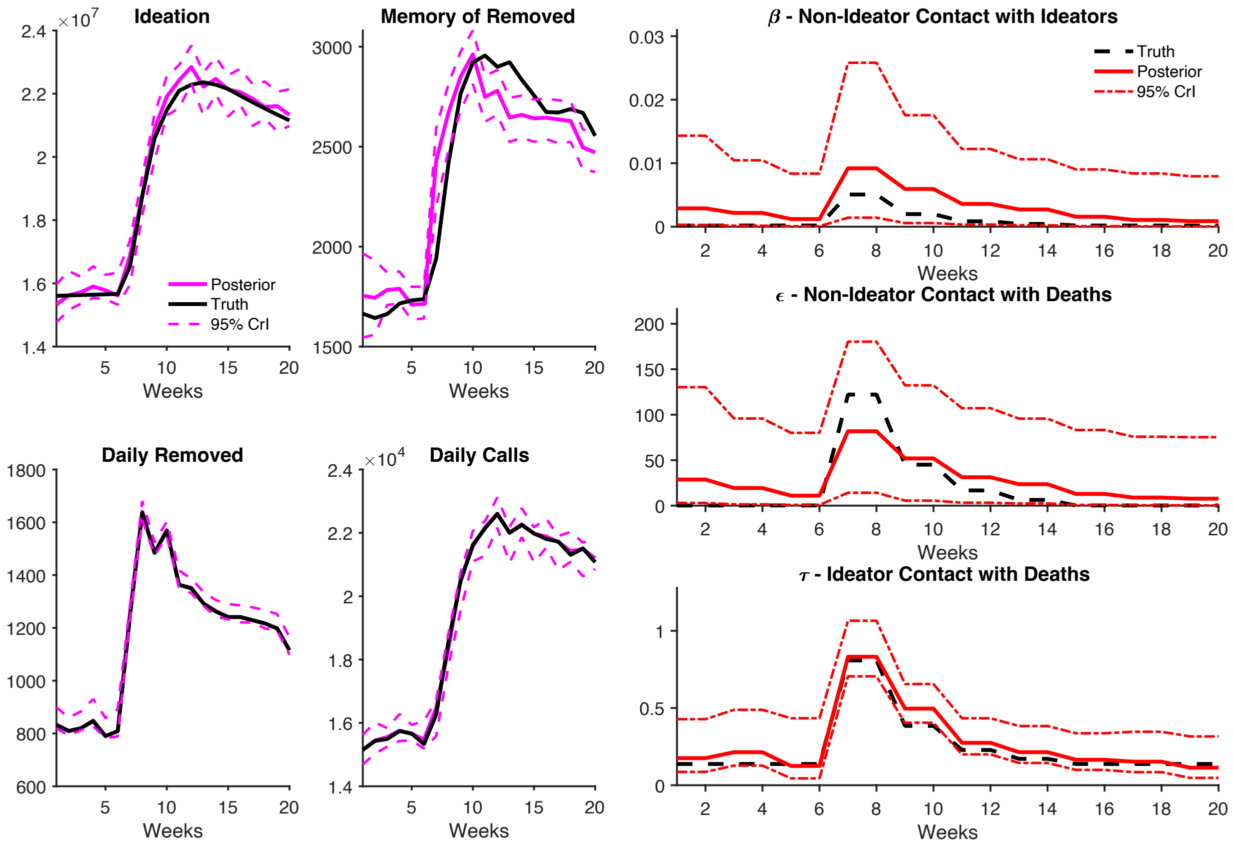

Figure S10. Synthetic test of the model-inference system. As in Figure S8, but with alternate values for the three nonlinear parameters (β, ϵ, τ).

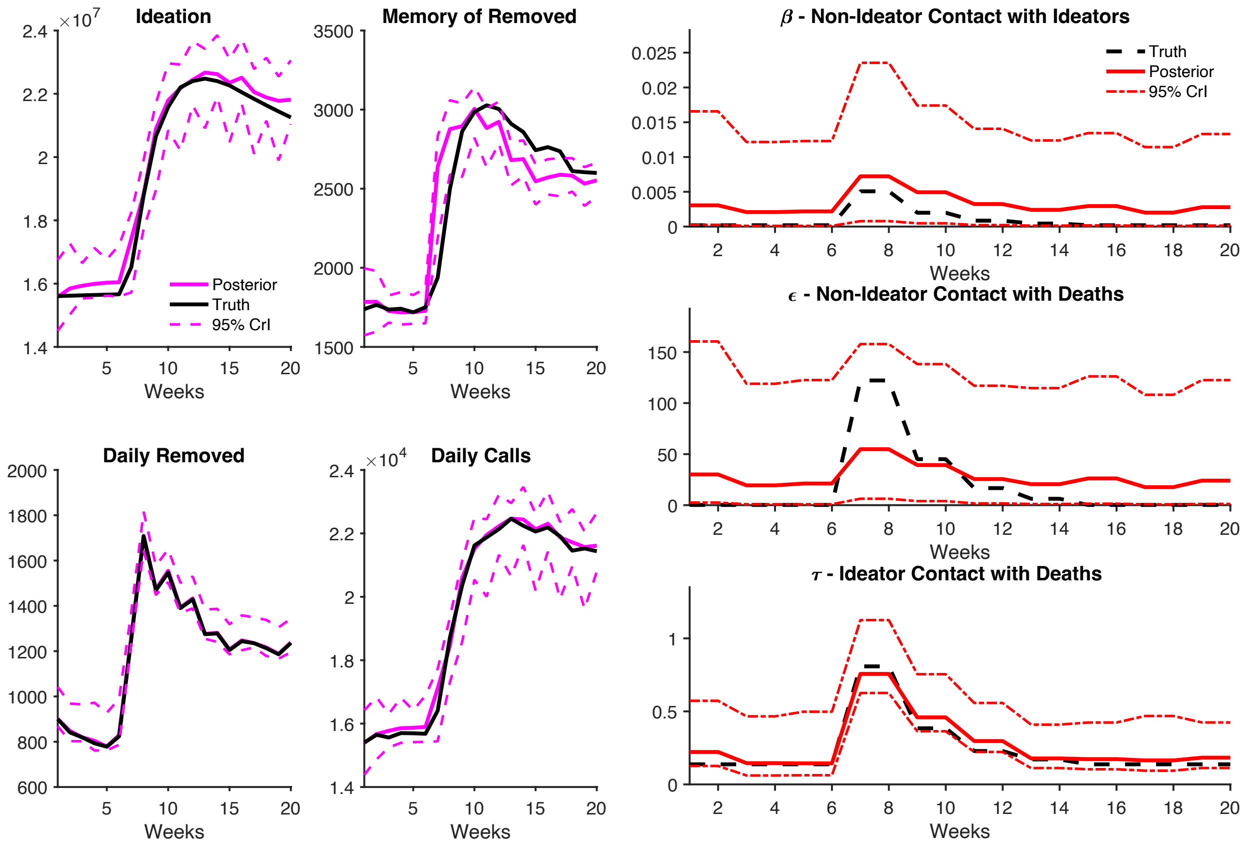

Figure S11. Synthetic test of the model-inference system. As in Figure S10, but with the observational error variance defined as $\sigma_{t,s}^2 = var(o_{ts})/20$.

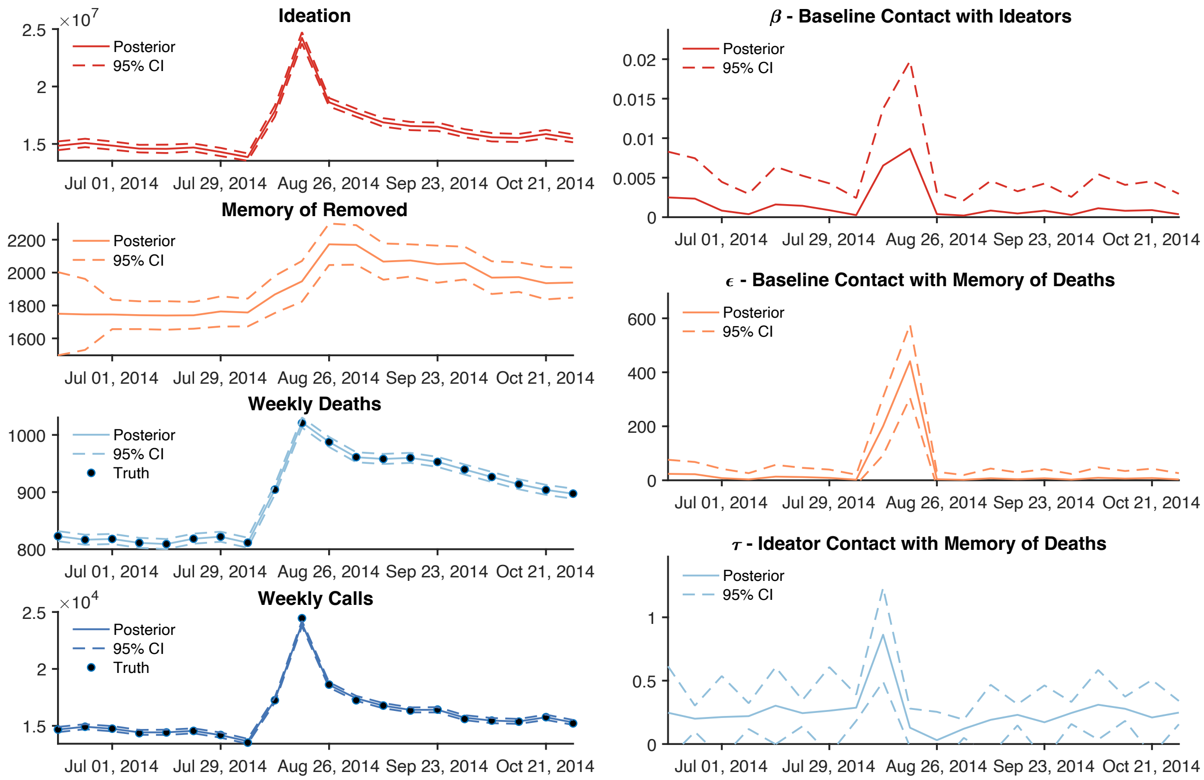

Figure S12. Simulation and inference for the 2014 RW suicide event. As for Figure 3 but with $\mu = 0.01/day$ and $\alpha = 0.00051/day$. The implementation of the IF-EAKF was run 20 times, each time with a 500-member ensemble. The estimates of all 10,000 simulations for each two-week time period were used to generate posterior estimates of the mean and distribution for the non-linear parameters and state variables. Left) Weekly fitting and simulation of the model-inference system to observed outcomes (red line). The blue line shows the mean fit; dashed blue lines are the 95% credible intervals. Right) Weekly estimates of model nonlinear parameters β, ϵ, τ. Solid lines are the mean estimate; dashed lines are the 95% credible intervals.

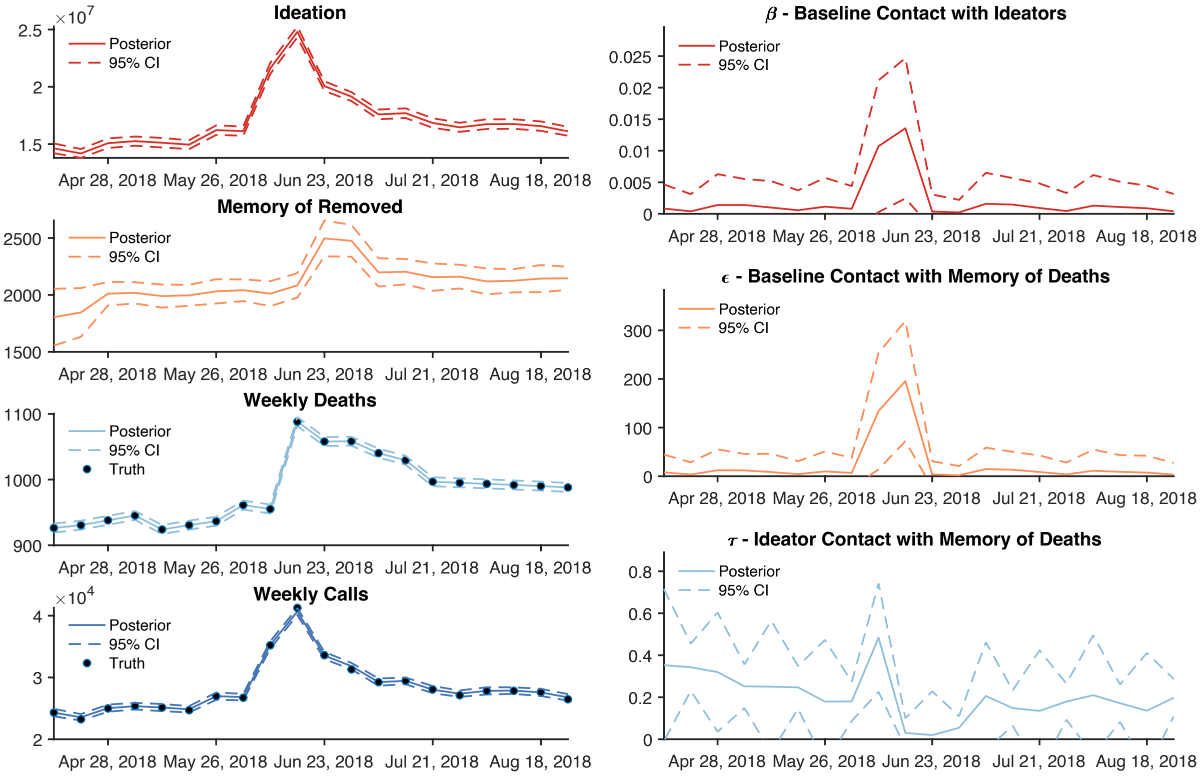

Figure S13. Simulation and inference for the 2018 KSAB suicide events. As for Figure 4 but with $\mu = 0.01/day$ and $\alpha = 0.00051/day$. The implementation of the IF-EAKF was run 20 times, each time with a 500-member ensemble. The estimates of all 10,000 simulations for each two-week time period were used to generate posterior estimates of the mean and distribution for the non-linear parameters and state variables. Left) Weekly fitting and simulation of the model-inference system to observed outcomes (red line). The blue line shows the mean fit; dashed blue lines are the 95% credible intervals. Right) Weekly estimates of model nonlinear parameters β, ϵ, τ. Solid lines are the mean estimate; dashed lines are the 95% credible intervals.

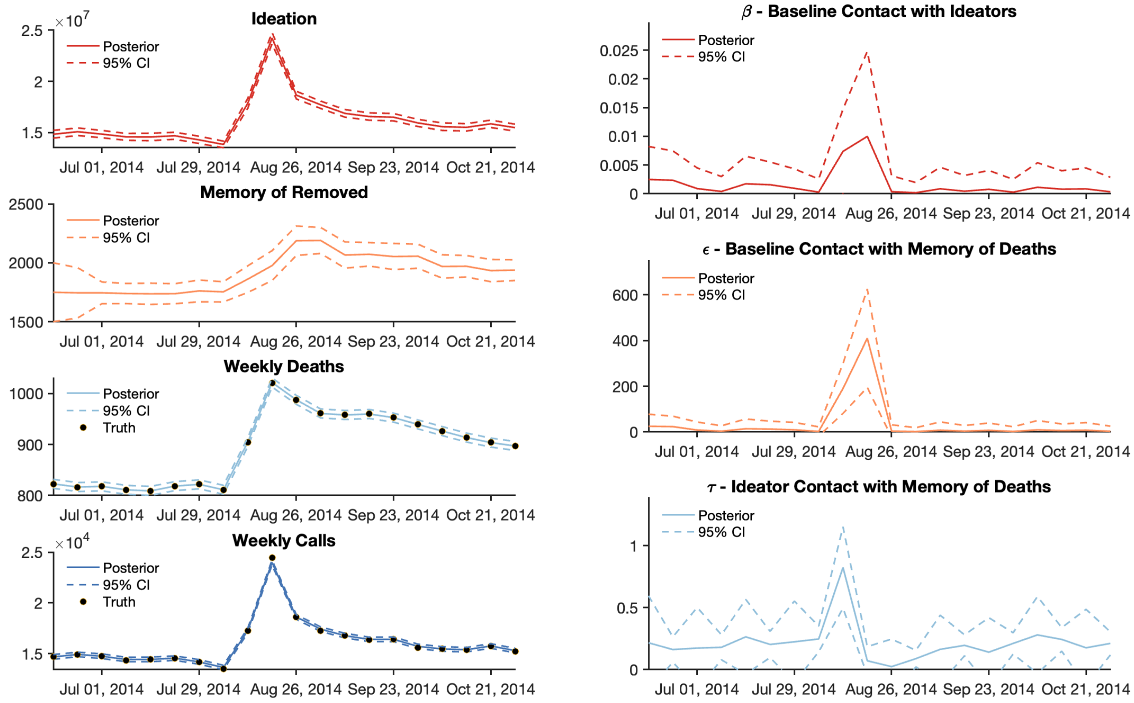

Figure S14. Simulation and inference for the 2014 RW suicide event. As for Figure 3 but with the assumption that 7% of suicide deaths are due to contagion ($\gamma = 6.93 \times 10^{-6}/day$). The implementation of the IF-EAKF was run 20 times, each time with a 500-member ensemble. The estimates of all 10,000 simulations for each two-week time period were used to generate posterior estimates of the mean and distribution for the non-linear parameters and state variables. Left) Weekly fitting and simulation of the model-inference system to observed outcomes (red line). The blue line shows the mean fit; dashed blue lines are the 95% credible intervals. Right) Weekly estimates of model nonlinear parameters β, ϵ, τ. Solid lines are the mean estimate; dashed lines are the 95% credible intervals.

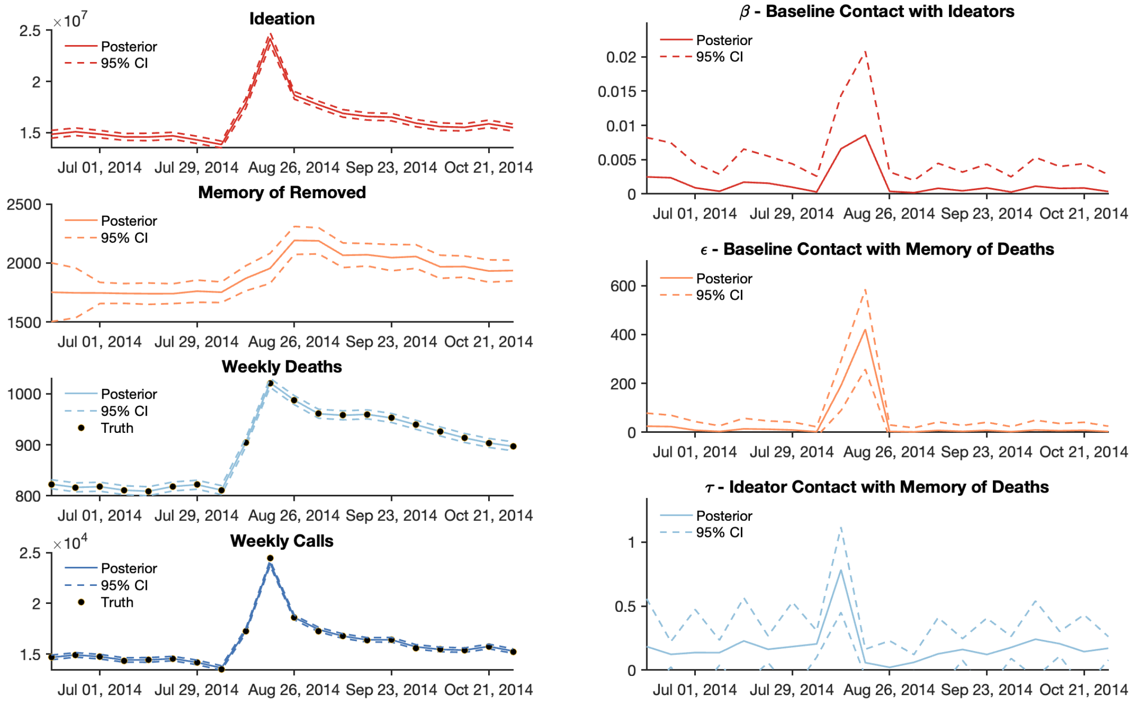

Figure S15. Simulation and inference for the 2014 RW suicide event. As for Figure 3 but with the assumption that 4% of suicide deaths are due to contagion ($\gamma = 7.16 \times 10^{-6}/day$). The implementation of the IF-EAKF was run 20 times, each time with a 500-member ensemble. The estimates of all 10,000 simulations for each two-week time period were used to generate posterior estimates of the mean and distribution for the non-linear parameters and state variables. Left) Weekly fitting and simulation of the model-inference system to observed outcomes (red line). The blue line shows the mean fit; dashed blue lines are the 95% credible intervals. Right) Weekly estimates of model nonlinear parameters β, ϵ, τ. Solid lines are the mean estimate; dashed lines are the 95% credible intervals.

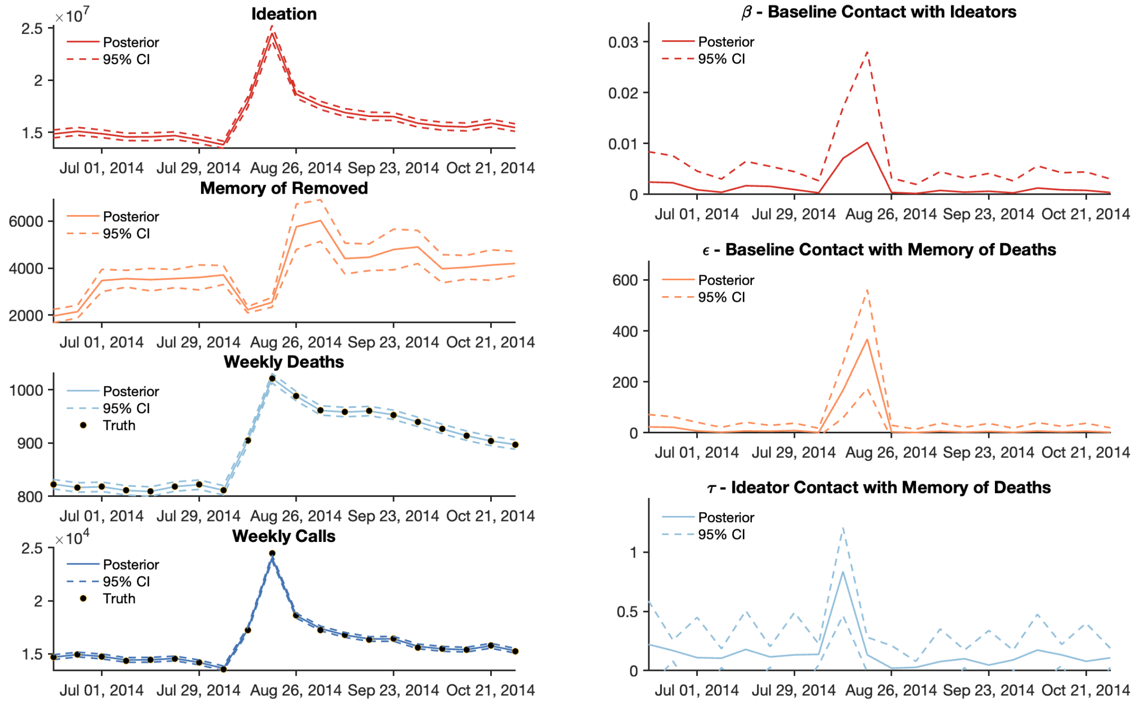

Figure S16. Simulation and inference for the 2014 RW suicide event. As for Figure 3 but with the assumption that $\kappa = 0.0333/day$). The implementation of the IF-EAKF was run 20 times, each time with a 500-member ensemble. The estimates of all 10,000 simulations for each two-week time period were used to generate posterior estimates of the mean and distribution for the non-linear parameters and state variables. Left) Weekly fitting and simulation of the model-inference system to observed outcomes (red line). The blue line shows the mean fit; dashed blue lines are the 95% credible intervals. Right) Weekly estimates of model nonlinear parameters β, ϵ, τ. Solid lines are the mean estimate; dashed lines are the 95% credible intervals.

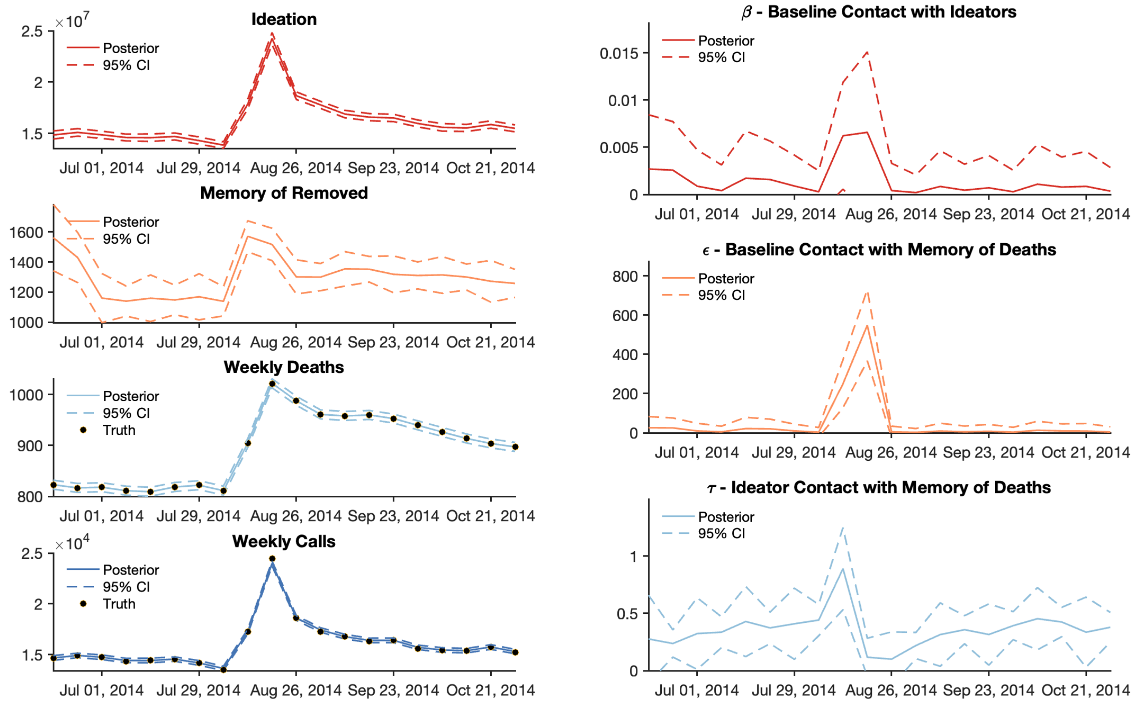

Figure S17. Simulation and inference for the 2014 RW suicide event. As for Figure 3 but with the assumption that $\kappa = 0.1/day$). The implementation of the IF-EAKF was run 20 times, each time with a 500-member ensemble. The estimates of all 10,000 simulations for each two-week time period were used to generate posterior estimates of the mean and distribution for the non-linear parameters and state variables. Left) Weekly fitting and simulation of the model-inference system to observed outcomes (red line). The blue line shows the mean fit; dashed blue lines are the 95% credible intervals. Right) Weekly estimates of model nonlinear parameters β, ϵ, τ. Solid lines are the mean estimate; dashed lines are the 95% credible intervals.

Table S1. Model state variable and parameter solutions for non-disease-free equilibria. Parenthetical values indicate alternate values used for sensitivity tests with higher μ. The call rate, ρ, the fraction of ideators calling Lifeline, is calculated from the steady state number of ideators and the observed average number of calls per week to Lifeline in the weeks leading up to each celebrity suicide.

Parameter	2013-2014 (Williams)	2017-2018 (Spade/Bourdain)	Source
Population ($N, people$)	318,000,000	327,000,000	Census Bureau
Ideators ($I_0, people$)	15,423,000	15,859,000	NHANES
Baseline Population ($B_0, people$)	303,577,000	303,141,000	Derived
Initial Awareness of Recent Suicides ($R_0, people$)	1724	1950	SI, Section 3
Birth Rate (b, day^{-1})	2.74×10^{-5}	2.74×10^{-5}	Census Bureau
Death Rate (d, day^{-1})	2.7×10^{-5}	2.7×10^{-5}	Census Bureau
Ideation Loss Rate (μ, day^{-1})	0.0049 (0.01)	0.0049 (0.01)	Zhang (2015)
Ideation Gain Rate (α, day^{-1})	0.00025 (0.00051)	0.00025 (0.00051)	Derived
Contact Rate of Non-Ideators with Ideators (β, day^{-1})	0.9423×10^{-4} (1.6131×10^{-4})	1.0207×10^{-4} (1.7517×10^{-4})	SI, Section 3
Contact Rate of Non-Ideators with Memory of Suicide Deaths (ϵ, day^{-1})	0.0937 (0.1603)	0.0916 (0.1583)	SI, Section 3
Contact Rate of Ideators with Memory of Suicide Deaths (τ, day^{-1})	0.1375	0.1375	SI, Section 3
Background Suicide Death Rate (γ, day^{-1})	6.71×10^{-6}	7.38×10^{-6}	SI, Section 3
Suicide Memory Loss Rate (κ, day^{-1})	0.0667	0.0667	SI, Section 3
Lifeline Call Rate (ρ, day^{-1})	1.42×10^{-4}	2.38×10^{-4}	Derived

Table S2. **Model initial conditions for state variables and nonlinear parameters.** Linear parameters were fixed as shown in Table S1.

Parameter	Initial Conditions
Ideators ($I_0, people$)	$U(1 \times 10^7, 2 \times 10^7)$
Baseline Population ($B_0, people$)	$N - I_0 - R_0$
Initial Awareness of Recent Suicides ($R_0, people$)	$U(1500{,}2000)$
Contact Rate of Non-Ideators with Ideators (β, day^{-1})	$U(1 \times 10^{-4}, 2.5 \times 10^{-4})$
Contact Rate of Non-Ideators with Memory of Suicide Deaths (ϵ, day^{-1})	$U(0{,}0.3)$
Contact Rate of Ideators with Memory of Suicide Deaths (τ, day^{-1})	$U(0{,}0.3)$

Table S3. **Key model assumptions.**

Assumption	Explanation
Constant birth and death rates	Model is designed to examine short-term responses to celebrity suicide events during which variations of birth and death are negligible
10% of suicide deaths derive from contagion	There is limited information on the percentage of suicide deaths due to contagion. 10% lies at the upper range of the estimate of Platt et al. (*27*)
Compartmental Model Structure	These models implicitly assume a perfectly-mixed population in which each member of the population is in equal contact with all other individuals. As for infectious diseases, this assumption, while unrealistic, enables simulation and estimation of population-mean properties without the need to specify local or individual connections or variability, which are largely unobserved.

Data S1. (separate file) File (Code_DataS1.zip) includes: Matlab code and input data files for running model-inference system (figure3_final_posting_copy.m; figure4_final_posting_copy.m; dlycalls13_14_notrend.mat; dlycalls17_18_notrend.mat; us_deaths_weekly_2013-14_noseas.mat; us_deaths_weekly_2017-18_noseas.mat; ReadMe file.

REFERENCES AND NOTES

1. S. Fazel, B. Runeson. Suicide. *N. Engl. J. Med.* **382**, 266–274 (2020).

2. M. K. Nock, I. Hwang, N. Sampson, R. C. Kessler, M. Angermeyer, A. Beautrais, G. Borges, E. Bromet, R. Bruffaerts, G. de Girolamo, R. de Graaf, S. Florescu, O. Gureje, J. M. Haro, C. Hu, Y. Huang, E. G. Karam, N. Kawakami, V. Kovess, D. Levinson, J. Posada-Villa, R. Sagar, T. Tomov, M. C. Viana, D. R. Williams. Cross-national analysis of the associations among mental disorders and suicidal behavior: Findings from the WHO world mental health surveys. *PLOS Med.* **6**, e1000123 (2009).

3. J. C. Franklin, J. D. Ribeiro, K. R. Fox, K. H. Bentley, E. M. Kleiman, X. Huang, K. M. Musacchio, A. C. Jaroszewski, B. P. Chang, M. K. Nock. Risk factors for suicidal thoughts and behaviors: A meta-analysis of 50 years of research. *Psychol. Bull.* **143**, 187–232 (2017).

4. M. Miller, D. Hemenway. Guns and suicide in the United States. *N. Engl. J. Med.* **359**, 989–991 (2008).

5. Q. Cheng, H. Li, V. Silenzio, E. D. Caine, Suicide contagion: A systematic review of definitions and research utility. *PLOS ONE* **9**, e108724 (2014).

6. M. Keeling, P. Rohani, *Modeling Infectious Diseases in Humans and Animals* (Princeton University Press, 2008).

7. D. P. Rosati, M. H. Woolhouse, B. M. Bolker, Modelling song popularity as a contagious process. **447**, 20210457 (2021).

8. N. T. M. Hill, L. S. Too, M. J. Spittal, J. Robinson, Understanding the characteristics and mechanisms underlying suicide clusters in Australian youth: A comparison of cluster detection methods. *Epidemiol. Psychiatr. Sci.* **29**, e151 (2020).

9. A. S. Mueller, S. Abrutyn, Suicidal disclosures among friends: Using social network data to understand suicide contagion. *J. Health Soc. Behav.* **56**, 131–148 (2015).

10. T. Niederkrotenthaler, S. Stack, B. Till, M. Sinyor, J. Pirkis, D. Garcia, I. R. Rockett, U. S. Tran, Association of increased youth suicides in the United States with the release of 13 Reasons Why. *JAMA Psychiatry* **76**, 933–40 (2019).

11. D. S. Fink, J. Santaella-Tenorio, K. M. Keyes, Increase in suicides the months after the death of Robin Williams in the US. *PLOS ONE* **13**, e0191405 (2018).

12. T. Niederkrotenthaler, M. Braun, J. Pirkis, B. Till, S. Stack, M. Sinyor, U. S. Tran, M. Voracek, Q. Cheng, F. Arendt, S. Scherr, P. S. F. Yip, M. J. Spittal, Association between suicide reporting in the media and suicide: Systematic review and meta-analysis. *Br. Med. J.* **368**, m575 (2020).

13. S. Scherr, A. Steinleitner, Zwischen dem werther- und papageno-effekt. *Nervenarzt*, **86**, 557–565 (2015).

14. M. Ueda, K. Mori, T. Matsubayashi, Y. Sawada, Tweeting celebrity suicides: Users' reaction to prominent suicide deaths on Twitter and subsequent increases in actual suicides. *Soc. Sci. Med.* **189**, 158–166 (2017).

15. B. R. Davis, R. J. Hardy, A suicide epidemic model. *Soc. Biol.* **33**, 291–300 (1986).

16. M. Ghosh, S. Das, P. Das, Dynamics and control of delayed rumor propagation through social networks. *J. Appl. Math. Comput.* **68**, 3011–3040 (2022).

17. M. Scata, A. Di Stefano, A. La Corte, P. Liò, Quantifying the propagation of distress and mental disorders in social networks. *Sci. Rep.* **8**, 5005 (2018).

18. D. Itzkoff, "Robin Williams, comic, Oscar-winning actor and TV alien, dies at 63," *New York Times*, 12 August 2014: https://nytimes.com/2014/08/12/movies/robin-williams-oscar-winning-comedian-dies-at-63.html.

19. B. Mueller, "Medical examiner says Kate Spade's death was a suicide," *New York Times*, 7 June 2018; https://nytimes.com/2018/06/07/nyregion/kate-spade-suicide.html.

20. M. Schreuer, “Sorrow and questions in a French village after Anthony Bourdain’s suicide,” *New York Times*, 9 June 2018; https://nytimes.com/2018/06/09/world/europe/anthony-bourdain-suicide-france.html.

21. 988 Suicide and Crisis Lifeline (2022); https://988lifeline.org.

22. National Vital Statistics System – Mortality Statistics, National Center for Health Statistics, U.S. Centers for Disease Control and Prevention (2022); https://cdc.gov/nchs/nvss/deaths.htm.

23. United States Census Bureau (2022); https://census.gov/data.html.

24. National Health and Nutrition Examination Survey: Questionnaires, datasets and related documentation, National Center for Health Statistics, U.S. Centers for Disease Control and Prevention (2022); https://cdc.gov/nchs/_nhanes/Default.aspx; https://cdc.gov/Nchs/Nhanes/2013-2014/DPQ_H.html.

25. D. J. Brody, L. A. Pratt, J. Hughes. Prevalence of depression among adults aged 20 and over: United States, 2013–2016. (NCHS Data Brief no. 303. National Center for Health Statistics, 2018).

26. Y. Zhang, P. S. F. Siu, S.-S. Chang, P. W. C. Wong, F. Y. W. Law, Association between changes in risk factor status and suicidal ideation incidence and recovery. *Crisis* **36**, 390–398 (2015).

27. J. M. Platt, J. R. Pamplin, C. Gimbrone, C. Rutherford, S. Kandula, M. Olfson, M. S. Gould, G. Martínez-Alés, J. Shaman, K. M. Keyes, Racial disparities in spatial and temporal youth suicide clusters. *J. Am. Acad. Child Adolesc. Psychiatry* **61**, 1131–1140.e5 (2022).

28. M. Sinyor, U. S. Tran, D. Garcia, B. Till, M. Voracek, T. Niederkrotenthaler. Suicide mortality in the United States following the suicides of Kate Spade and Anthony Bourdain. *Aust. N. Z. J. Psychiatry* **55**, 613–619 (2021).

29. M. Sinyor, A. Schaffer, Y. Nishikawa, D. A. Redelmeier, T. Niederkrotenthaler, J. Sareen, A. J. Levitt, A. Kiss, J. Pirkis, The association between suicide deaths and putatively harmful and protective factors in media reports. *CMAJ* **190**, e900–e907 (2018).

30. C. C. Sorensen, M. Lien, V. Harrison, J. J. Donoghue, J. S. Kapur, S. H. Kim, N. T. Tran, S. V. Joshi, S. G. Patel, The tool for evaluating media portrayals of suicide (TEMPOS): Development and application of a novel rating scale to reduce suicide contagion. *Int. J. Environ. Res. Public Health* **19**, 2994 (2022).

31. N. T. M. Hill, J. Robinson, J. Pirkis, K. Andriessen, K. Krysinska, A. Payne, A. Boland, A. Clarke, A. Milner, K. Witt, S. Krohn, A. Lampit, Association of suicidal behavior with exposure to suicide and suicide attempt: A systematic review and multilevel meta-analysis. *PLOS Med.* **17**, e1003074 (2020).

32. T. A. B. Snijders, G. G. van de Bunt, C. E. G. Steglich, Introduction to stochastic actor-based models for network dynamics. *Soc. Networks* **32**, 44–60 (2010).

33. T. A. B. Snijders, Stochastic actor-oriented models for network dynamics. *Annu. Rev. Stat. Appl.* **4**, 343–363 (2017).

34. W. J. Burk, C. E. G. Steglich, T. A. B. Snijders, Beyond dyadic interdependence: Actor-oriented models for co-evolving social networks and individual behaviors. *Int. J. Behav. Dev.* **31**, 397–404 (2007).

35. M. Sentse, J. K. Dijkstra, M. Salmivalli, A. H. N. Cillessen, The dynamics of Friendships and victimization in adolescence: A longitudinal social network perspective. *Aggress. Behav.*, **39**, 229–238 (2013).

36. C. Berger, M. C. Gremmen, D. Palacios, E. Franco, "Would you be my friend?": Friendship selection and contagion processes of early adolescents who experience victimization. *J. Early Adolesc.*, **39**, 1286–1310 (2019).

37. R. Whitley, D. S. Fink, J. Santaella-Tenorio, K. M. Keyes, Suicide mortality in Canada after the death of Robin Williams, in the context of high-fidelity to suicide reporting guidelines in the Canadian media. *Can. J. Psychiatry*, **64**, 805–812 (2019).

38. J. Pirkis, D. Currier, L. S. Too, M. Bryant, S. Bartlett, M. Sinyor, M. J. Spittal, Suicides in Australia following media reports of the death of Robin Williams. *Aust. N. Z. J. Psychiatry* **54**, 99–104 (2020).

39. A. Pitman, D. S. Fink, R. Whitley, Patterns of suicide mortality in England and Wales before and after the suicide of the actor Robin Williams. *Soc. Psychiatry Psychiatr. Epidemiol.* **56**, 1801–1808 (2021).

40. World Health Organization, *International Statistical Classification Of Diseases and Related Health Problems, 10th revision*. (WHO, 2022).

41. E. L. Ionides, C. Bretó, A. A. King, Inference for nonlinear dynamical systems. *Proc. Natl. Acad. Sci. U.S.A.* **103**, 18438–18443 (2006).

42. J. L. Anderson, An ensemble adjustment Kalman filter for data assimilation. *Mon. Weather Rev.* **129**, 2884–2903 (2001).

43. A. A. King, E. L. Ionides, M. Pascual, M. J. Bouma, Inapparent infections and cholera dynamics. *Nature* **454**, 877–880 (2008).

44. D. He, E. L. Ionides, A. A. King. Plug-and-play inference for disease dynamics: Measles in large and small populations as a case study. *J. R. Soc. Interface* **7**, 271–283 (2010).

45. S. Pei, S. Kandula, W. Yang, J. Shaman, Forecasting the spatial transmission of influenza in the United States. *Proc. Natl. Acad. Sci. U.S.A.* **115**, 2752–2757 (2018).

46. R. Li, S. Pei, B. Chen, Y. Song, T. Zhang, W. Yang, J. Shaman, Substantial undocumented infection facilitates the rapid dissemination of novel coronavirus (SARS-CoV-2). *Science* **368**, 489–493 (2020).

47. S. Pei**,** T. K. Yamana**,** S. Kandula, M. Galanti, J. Shaman, Burden and characteristics of COVID-19 in the United States during 2020. *Nature* **598**, 338–341 (2021).

48. T. Schneider, O. R. A. Dunbar, J. Wu, L. Boettcher, D. Burov, A. Garbuno-Inigo, G. L. Wagner, S. Pei, C. Daraio, R. Ferrari, J. Shaman, Epidemic management and control through risk-dependent individual contact interventions. *PLOS Comp. Biol.* **18**, e1010171 (2022).

49. C. Snyder, T. Bengtsson, P. Bickel, J. Anderson, Obstacles to high-dimensional particle filtering. *Mon. Weather Rev.* **136**, 4629–4640 (2008).

50. J. S. Whitaker, T. M. Hamill, Ensemble data assimilation without perturbed observations. *Mon. Weather Rev.* **130**, 1913–1924 (2002).

51. W. Yang, J. Shaman, A simple modification to improving inference for non-linear dynamical systems. arXiv: 1403.6804 [stat.ME] (2014). https://doi.org/10.48550/arXiv.1403.6804.
